# Extracellular vesicle-mediated transfer of processed and functional RNY5 RNA

**DOI:** 10.1261/rna.053629.115

**Published:** 2015-11

**Authors:** Sudipto K. Chakrabortty, Ashwin Prakash, Gal Nechooshtan, Stephen Hearn, Thomas R. Gingeras

**Affiliations:** Cold Spring Harbor Laboratory, Cold Spring Harbor, New York 11724, USA

**Keywords:** extracellular vesicles, exosomes, RNY5, cancer microenvironment

## Abstract

Extracellular vesicles (EVs) have been proposed as a means to promote intercellular communication. We show that when human primary cells are exposed to cancer cell EVs, rapid cell death of the primary cells is observed, while cancer cells treated with primary or cancer cell EVs do not display this response. The active agents that trigger cell death are 29- to 31-nucleotide (nt) or 22- to 23-nt processed fragments of an 83-nt primary transcript of the human *RNY5* gene that are highly likely to be formed within the EVs. Primary cells treated with either cancer cell EVs, deproteinized total RNA from either primary or cancer cell EVs, or synthetic versions of 31- and 23-nt fragments trigger rapid cell death in a dose-dependent manner. The transfer of processed RNY5 fragments through EVs may reflect a novel strategy used by cancer cells toward the establishment of a favorable microenvironment for their proliferation and invasion.

## INTRODUCTION

Since the observation that various types of RNAs are part of the cargo of extracellular vesicles (EVs) (Ratajczak et al. 2006; Valadi et al. 2007; Skog et al. 2008), numerous efforts have been made to catalog RNA cargos and determine whether these RNAs are biologically functional (Luo et al. 2009; Koh et al. 2010; Nolte-’t Hoen et al. 2012; Zhou et al. 2012). The question of the functionality of the RNA cargos has been made complicated by the observation that a large proportion of the detected RNA biotypes are represented by a mixture of full-length and shorter fragments (Tuck and Tollervey 2011; Dhahbi et al. 2013a,b; Vojtech et al. 2014). With perhaps the exception of micro- (mi) RNA cargos, the issue of the functionality of RNAs released and carried by EVs remains largely unresolved. We describe a study of the human (h)Y RNA family that seeks to address this issue.

The *hY* RNA family consists of four genes (*RNY1*, *RNY3*, *RNY4*, *RNY5*) that are transcribed by RNA polymerase III, whose primary transcripts range in length from ∼83–112 nucleotides (nt) (Hendrick et al. 1981; Wolin and Steitz 1983; O'Brien and Harley 1990). The evolutionary conservation of this gene family is underscored by the sequence similarity of these RNA genes seen in all vertebrates and more recently in invertebrates (Sim and Wolin 2011). Additionally, the presence of 966 *hY* RNA pseudogenes, of which *hY5* has eight in the human genome, also underscores their long evolutionary heritage (Perreault et al. 2005, 2007). An understanding of the underlying biological roles of this class of RNAs developed slowly since their discovery in 1981 (Hendrick et al. 1981). At the outset, the associations of the *hY* RNAs with both Ro60 and La proteins in ribonucleoprotein complexes found in normal and in systemic lupus erythematosus and Sjogren's syndrome samples (Lerner et al. 1981) were the first indications of possible biological roles of these short (s)RNAs. Since these original observations, multiple descriptions of other ribonucleoprotein complexes involving Y RNAs have been described, prompting the hypothesis that *Y-*RNAs may have multiple functions based on the protein partners present in the complexes (Sim et al. 2009). More recently, support for this hypothesis has been provided by reports that cellular *Y*-RNAs have specific functional roles including forming part of the initiation of DNA replication complex (Christov et al. 2006; Gardiner et al. 2009), the chaperoning of misfolded RNAs (Chen et al. 2003; Gardiner et al. 2009), and assisting in the quality control of *5S* ribosomal RNAs (Hogg and Collins 2007). Correlated with each of these functional roles has been the identification of a variety of distinct proteins associated with the *Y*-RNAs involved. Finally, *hY* RNAs are significantly up-regulated between five- to 13-fold in human cancer tissues compared to normal tissues (Langley et al. 2010).

In addition to the presence of the full-length *hY* RNAs, fragments of each of the four *hY* RNAs have been found inside and outside of cells. Northern analyses of human Jurkat T-lymphocyte cell line induced into apoptosis showed rapid Ago 2-independent processing of the *hY* RNAs into fragments of multiple lengths (Nicolas et al. 2012). Fragments of *hY* RNAs have also been detected outside of cells in healthy human serum and plasma isolates, using RNA sequencing (RNA-seq) (Dhahbi et al. 2013a). While the lengths of the processed RNAs observed outside of cells were seen to be similar to that observed within cells, ∼95% of the sequences detected were mapped to *hY4* with only a minor fraction mapping to the other three *hY* RNAs. The detected fragments consisted of the 5′ end sequences of each of the full-length *hY*RNA transcripts but were determined not to be cargos of EVs. It has been conjectured that they are part of circulating ribonucleoprotein complexes. Extracellular fragments of *hY* RNAs have also been found in EVs isolated from human semen (Vojtech et al. 2014) and mouse co-cultured dendritic-T cells (Nolte-’t Hoen et al. 2012). A 30- to 33-nt RNY4 fragment and a 28-nt fragment from unspecified mouse YRNA, both starting from the 5′ end of the annotated genes, have also been detected.

Although various members of the *hY* RNA families have been observed to be selectively enriched and made part of EV RNA cargos, a comprehensive study of the relationship of the full-length primary transcript *hY* RNAs to processed forms and if any of these forms are biologically active has yet to be carried out. Additionally, any differences in the processed versus the primary transcripts for the *Y*-RNAs found in the EVs released by different types of normal and transformed cells have yet to be reported. This study explores the processing and transfer of specific RNY5 fragments within EVs derived from cancer cells and their cellular phenotype associated with induction of primary cell death.

## RESULTS

### Isolation, quantification, and characterization of EV RNA cargos of primary and cancer cell lines

**FIGURE 1.**
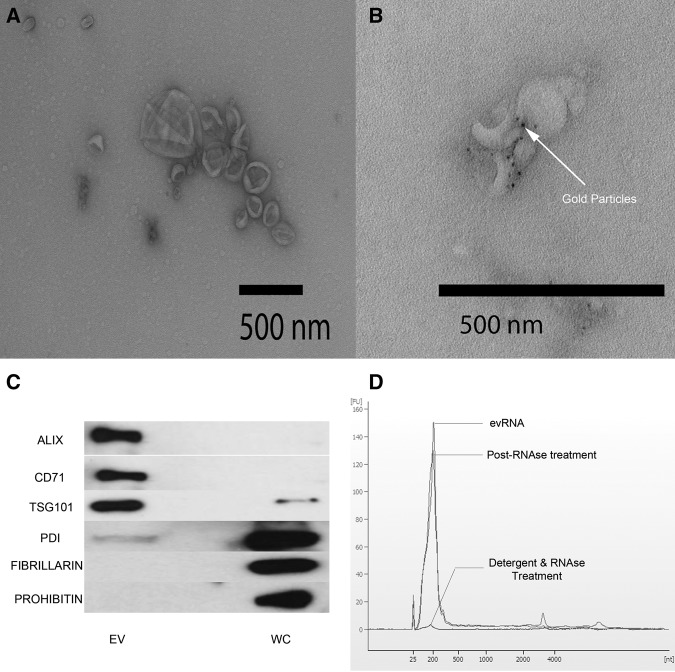
Validation of purification of extracellular vesicles (EVs). (*A*) Transmission electron microscopy image of K562 EVs after negative staining shows classic cup-shaped vesicles that are on average smaller than 200 nm. (*B*) Immuno-electron microscopy image of purified EVs labeled with anti-CD81 (mouse mAb) and detected by goat anti-mouse IgG secondary conjugated with 5 nm gold. Dark spots on the image are the electron dense gold elements conjugate to IgG secondary antibody. (*C*) Bioanalyzer RNA profile (RNA Pico-chip) of untreated EVs (red), RNA profile of EVs treated with RNase (green), and RNA profile of EVs treated with detergent and RNase (blue). (*X*-axis) Nucleotide lengths; (*y*-axis) fluorescent units. (*D*) Western blot analysis of proteins from K562 EVs and whole cell. Proteins selected for detection were previously identified to be enriched in EV or whole cell. (EV enriched) ALIX (*PDCD6IP* gene), CD71 (*TfR1* gene), TSG101 (*TSG101* gene). (Whole cell) PDI (*PDI* gene), FIBRILLARIN (*FBL* gene), PROHIBITIN (*PHB* gene).

Enriched preparations of EVs were carried out using the modified version of the protocol first described by Thery et al. (2006) (Supplemental Fig. S1A). Verification of the isolation and enrichment of EVs compared with the cells of origin (K562 myelogenous leukemia and BJ primary fibroblast) was carried out using three methods: transmission electron (Fig. 1A) and immuno-electron micrographic techniques (Fig. 1B) and Western blot analyses of the EV-specific membrane proteins compared with several cellular protein markers (Fig. 1C). The determination that the detected RNAs are cargos of the EVs rather than an artifact associated with EV purification was made treatment of preparation of EVs prior to RNA isolation with RNase A and T1 and compared with RNA isolated from untreated EVs as well as EVs treated with detergent followed by RNase (Fig. 1D). These results indicate that the RNAs isolated from EVs were internalized within vesicles and thus protected from nuclease attack. Using a nanoparticle tracking technology (Nanosight), the number of EVs isolated from cultured 10^8^ K562 cells was very conservatively estimated to be approximately 1.1 × 10^11^ (Supplemental Fig. S1B; Supplemental Table S1). Of all cell lines studied, K562 cells were observed to have the most EVs released. A more typical EV production from the same number of cells is exemplified by the BJ cell lines of approximately 4.8 × 10^9^. However, the approximately 23-fold difference in EV number is reduced to a two- to fourfold difference in the amounts of RNA isolated from the EVs of each cell type (Supplemental Table S1). While this difference could be attributable to differences in amounts of RNA in the EVs of the two cell types, it is more likely the imprecision of EV counting that is attributable to the documented clumping of EVs.

**FIGURE 2.**
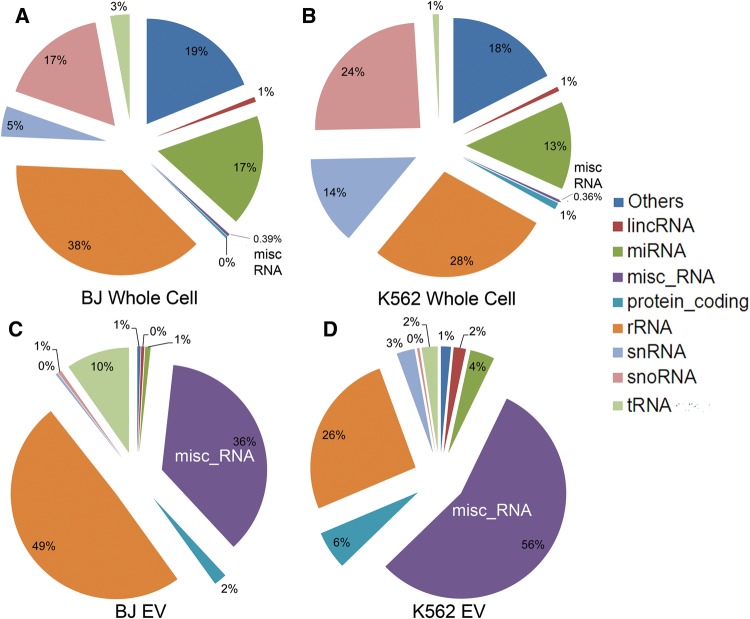
Pie charts representing the relative abundance of families of RNA within BJ whole cell (*A*), K562 whole cell (*B*), BJ EV (*C*), and K562 EV (*D*). The group labeled as “Others” in the pie charts is representative of reads derived from several Gencode annotation categories such as pseudogenes, antisense intronic, mitochondrial t-RNA, vault RNA, immunoglobulin genes, etc.

To study the RNA content of isolated EVs, we carried out an RNA-seq profile analysis on replicates of whole cells and EV cargos derived from K562 (myelogenous leukemia) and BJ (foreskin fibroblast) cells. Profiles obtained from both cell lines and enriched EVs were highly reproducible (Supplemental Fig. S2A,B). However, a low degree of correlation between RNA profiles in EVs and their source cells was readily evident. A detailed quantification of annotated sRNAs (reads per million [rpm]) isolated from BJ and K562 whole cells (Fig. 2A,B) indicated a predominance of rRNA, snoRNA, and miRNAs. In contrast, the relative distribution of sRNAs in EVs from the same cells indicates almost a considerable enrichment of the miscellaneous RNA (miscRNA) group and predominance of rRNA and tRNA (Fig. 2C,D). A comparison of the relative abundance of sRNA families between source cells and their EVs specifically highlights the enrichment of genes within the miscRNA group, consisting of several families of sRNAs—*small Cajal body* (*sca*), *Y-*RNA and *vault* (*vt*) RNAs (Supplemental Fig. S3). *RNY5* was the most abundant miscRNA gene present in EVs, composing 35% of all sRNAs in BJ EVs and 48% in K562 EVs. In contrast, *RNY5* accounts for only 0.1% and 0.2% of all reads from sRNAs within BJ and K562 whole cells, respectively. In EVs from both BJ and K562, the *RNY5* gene contributes >89% of the reads from miscRNA, whereas in whole cells it constitutes only 40% of miscRNA reads, emphasizing the particular enrichment of this gene within EVs. Enrichment levels of *RNY5* in EVs compared with whole-cell RNAs from BJ and K562 were 196- and 68-fold, respectively.

### Processing of *RNY*5 RNAs in EVs

**FIGURE 3.**
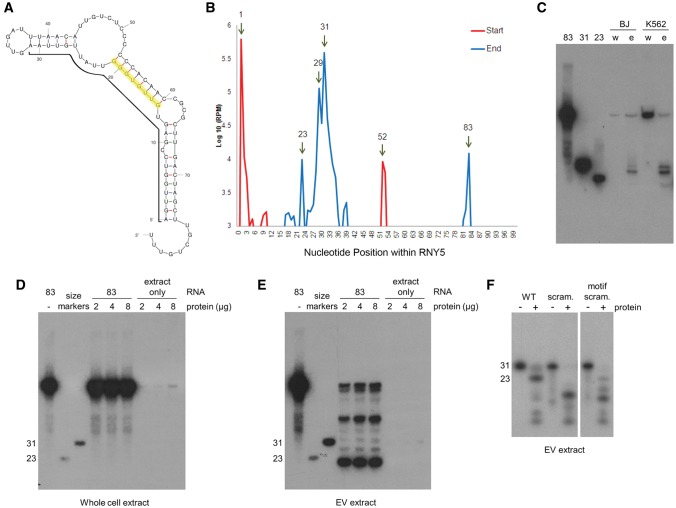
Fragmentation patterns of RNY5. (*A*) Full-length RNY5 structure. The structure was drawn using Mfold according to data from van Gelder et al. (1994). Bold line indicates the 5′ 31-nt processed product and the 8-nt motif is highlighted. (*B*) Graph depicting the most frequent (>1000 reads per million) start and stop positions of reads mapping to the human RNY5 gene. The most frequent start positions marked as the 5′ start position of the RNY5 annotation and position 52 of the annotation. The most frequent stop positions are 23, 29, and 31 for the reads, which start at the 5′ end of the RNY5 gene, and position 83 which has reads starting at 52 and also some reads that start at position 1. (*C*) Northern blot of RNY5 RNA purified from K562, BJ cells, and EVs. Synthetic versions of Y5 processing products were used as size markers. RNA was detected by a probe complementary to the 5′ 31-nt processed product. (w) Whole-cell RNA, (e) EV RNA. (*D*,*E*) In vitro processing of RNY5. Synthetic full-length RNY5 was incubated for 30 min at 37° with 0, 2, 4, or 8 µg of K562 whole cell (*D*) or EV (*E*) protein extract. Samples containing only the extracts and treated identically were used to control for the existence of Y5 RNA in protein extracts. Detection was done as in *C*. Note that 23- and 31-nt size markers are not equimolar. (*F*) In vitro processing of Y5 5′ 31-mer variants. Wild-type (WT), scrambled (scram.), and 8-nt motif scrambled (motif scram.) versions of the Y5 5′ 31-mer were radioactively end-labeled and incubated with K562 EV protein extract for 2 h at 37°.

In the EVs, using RNA-seq data, the 83-nt *RNY5* primary transcript (Fig. 3A) was detected as well as shorter products of 23, 29, and 31 nt in length, with start and end positions for each of these forms located at the 5′ end of the Gencode gene annotation (Fig. 3B). Additionally, a separate 31-nt product mapping between nucleotide positions 51 and 83 (3′end of RNY5) of the primary transcript was observed, which is partially complementary to the 31-nt 5′ fragment (Fig. 3A).

Northern hybridization analyses using a probe complementary to the first 31 nt of the *RNY5* showed that the form of *RNY5* present in the whole cell was the full-length 83-nt transcript (Fig. 3C). While the RNA extracted from EVs contained the 83-nt transcript, it was highly enriched for the 29- to 31-nt forms, as well as a modest amount of a 23-nt product, which is in agreement with the RNA-seq results observed for the EV RNAs (Fig. 3B). Similarly, the 31-nt 3′RNY5 processed transcript can also be detected within EVs (data not shown).

To further investigate the processing of *RNY*5 seen in the EVs, we incubated a synthetic form of the 83-nt *RNY5* transcript with K562 whole-cell and EV protein extracts, followed with detection by Northern analysis. We found that synthetic copies of the 83-nt *RNY5* incubated with K562 whole-cell extracts exhibit no detectable processing (Fig. 3D), whereas incubation with K562 EV extracts leads to dose-dependent formation of all processed forms (23, 29, 31 nt) detected in vivo (Fig. 3E)*.* Additionally, a prominent *RNY5* processed species larger than 31 nt is detected. The altered ratios of processed products and the appearance of a larger species in vitro may well be caused by the different conditions in an in vitro reaction (Fig. 3E). Treatment of the synthetic version of the 31-nt RNA with K562 EV extract produced the same 23-nt product as seen using the 83-nt substrate (Fig. 3F), confirming that the 23-nt product can be produced from either an 83- or 31-nt substrate. However, when a shuffled version of the 31-nt RNA (see Materials and Methods section) was treated with EV extract, no 23-nt product is observed, demonstrating the sequence specificity of the processing activity of the EV extract (Fig. 3F).

Gardiner et al. (2009) reported that a conserved double-stranded sequence motif in the upper stem of all vertebrate *Y*-RNAs correlated with their participation in initiating DNA replication. Each of the products processed from the 5′ side of *RNY*5 in vivo and in vitro contains a single-stranded version of this motif. The motif is 8 nt long (5′-GUUGUGGG-3′) extending from nucleotides 14–21 of *RNY5* (Fig. 3A). An alternate form of the 31-nt substrate carrying a shuffled motif only exhibits residual processing into a 23-nt product (Fig. 3F), underscoring the importance of the motif for processing of *RNY5* transcripts into the smaller fragments.

### Intercellular transfer and subcellular localization of EVs and their RNA cargos

The transfer of EVs and their molecular cargos from one cell type to another has previously been documented by use of both microscopic and molecular methods (Lee et al. 2012). We have extended these studies by monitoring the transfer of EVs between K562 and BJ cells and between K562 and two mouse cell lines (3T3 and HB4). The goals of these experiments were to confirm the transfer of RNA content of EVs from one cell type to another in a species-independent manner and to identify the subcellular localization and kinetics of the transferred EVs and RNA contents.

K562 EVs were first labeled with the lipid dye PKH67 (see Supplemental Material) after isolation. Following exposure of human BJ cells to labeled EVs, the EVs were found to be localized almost exclusively in the cytosol (Supplemental Fig. S4A). To monitor the transfer of EV RNA, K562 cells were metabolically labeled with 5′ ethynyl uridine, and EVs were isolated. Transfer of labeled RNA contained in EVs was monitored after entry into mouse 3T3 cells. The localization of the labeled RNAs was also found to be primarily cytoplasmic (Supplemental Fig. S4B). The same cytosolic localization was observed when primary human fibroblasts (BJ cells) were transfected with synthetic 31-nt oligonucleotides versions of *RNY5* via lipofection (Supplemental Fig. S4C). These data also point to a lack of cell-type and species specificity in the transfer of the EVs. This former property was also observed with EVs from multiple human cell types transferred into different recipient cell lines (data not shown).

The kinetics of intercellular transfer of EV RNAs was studied by treating mouse HB4 cells with EVs from human K562 cells followed by RNA-seq analysis. Mouse cells were chosen for this experiment as a recipient cell type because of the absence of the *RNY5* gene in the mouse genome, allowing for the unambiguous monitoring of human *RNY5* transcripts. A temporal study lasting 24 h revealed that maximum levels of *RNY5* were achieved by 12 h post-exposure followed by a progressive decrease in *RNY5* levels (Supplemental Fig. S4D).

### Biological phenotypes produced by EVs and *hY5* RNA fragments

**FIGURE 4.**
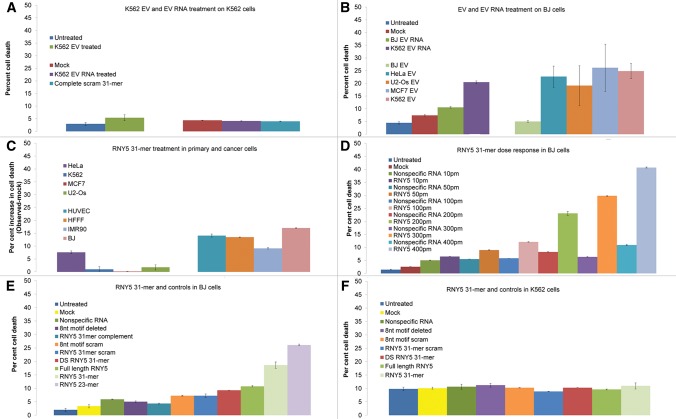
Quantification of cell death by flow cytometry. YO-PRO-1 and Hoechst33342 dyes were used for quantification of cell death. *Y*-axis indicates the percent of cell death indicated by YO-PRO-1 and Hoechst33342 double positive cells. The mean of duplicates is presented with error bars indicating variation from mean. (*A*) Levels of cell death in K562 cells when treated with EVs and EV RNA. *Y*-axis indicates percent cell death observed. The following treatments are presented: (Blue) untreated: K562 cells without any treatment; (green) K562 EV treated: K562 cells incubated with K562 EVs; (red) mock: K562 cells with Lipofectamine treated only (no RNA); (purple) K562 EV RNA treated: K562 cells treated with K562 EV RNA; (turquoise) complete scram 31-mer: k562 cells treated with 31-nt scrambled sequence. (*B*) Levels of cell death in BJ cells when treated with EVs and EV RNA. *Y*-axis indicates percent cell death observed. The following treatments are presented: (Blue) untreated: BJ cells without any treatment; (red) mock: BJ cells with lipofectamine treated only (no RNA); (green) BJ EV RNA: BJ cells transfected with BJ EV RNA; (purple) K562 EV RNA: BJ cells treated with K562 EV RNA; (light green) BJ EV: BJ cells incubated with BJ EVs; (turquoise) HeLa EV: BJ cells incubated with HeLa EVs; (orange) U2-Os EV: BJ cells incubated with U2-Os EVs; (light blue) MCF7 EV: BJ cells incubated with MCF7 EVs; (mauve) K562 EV: BJ cells incubated with K562 EVs. (*C*) Generality of RNY5 31-mer-induced cell-death phenotype. Bars indicate the net increase in cell death normalized to levels of cell death from mock treatment in each cell type. Four cancer cell lines including (blue) K562 (chronic myelogenous leukemia); (purple) HeLa (cervical adenocarcinoma); (red) MCF7 (breast adenocarcinoma); (green) U2-Os (osteosarcoma), and four primary cells including (light mauve) BJ (normal skin fibroblasts); (turquoise) HUVEC (normal human umbilical vein endothelial cell); (light blue) IMR90 (normal human lung fibroblasts); and (orange) HFFF (normal human fetal foreskin fibroblasts) were transfected with RNY5 31-mer. One hundred picomoles of RNY5 was used for each transfection, except (orange) HFFF, where 200 pmol of RNY5 31-mer was used. (*D*) Dose response curve of RNY5 31-mer-induced cell-death phenotype in BJ cells. The bars represent the percent of cell death when BJ cells are treated with increasing dose (10, 50, 100, 200, 300, and 400 pmol) of RNY5 31-mer or nonspecific RNA. AllStars negative control RNA (Qiagen) was used as a nonspecific RNA control. The levels of cell death in Untreated or Mock treated (Lipofectamine only) BJ cells are also indicated. (*E*) Levels of cell death in BJ cells from 100 pmol of synthetic RNA oligonucleotides transfection. *Y*-axis indicates the percent cell death. The synthetic RNA oligonucleotides used for transfection are as follows: (Blue) untreated: BJ cells without any treatment; (yellow) mock: BJ cells with Lipofectamine treated only (no RNA); (green) nonspecific RNA: nonspecific RNA control (AllStars negative control siRNA); (purple) 8-nt motif deleted: RNY5 sequence with nucleotides 14–21 motif deleted; (turquoise) RNY5 31-mer complement: 31-nt RNY5 3′ side fragment; (light orange) 8-nt motif scrambled: RNY5 31-mer sequence with nucleotides 14–21 scrambled; (blue) RNY5 31-mer scram: 31-nt completely scrambled sequence; (orange) DS RNY5 31-mer, double-stranded RNY5 31-mer duplex; (green) full-length RNY5: RNY5 83-mer full-length sequence; (light green) RNY5 31-mer: 5′ RNY5 31-nt fragment; (light purple) RNY5 23-mer: 5′ side RNY5 23-nt fragment. (*F*) Levels of cell death observed in K562 cells from 100 pmol of synthetic RNA oligonucleotides transfection. *Y*-axis indicates percent cell death. The synthetic RNA oligonucleotides used for transfection are as follows: (Blue) untreated: K562 cells without any treatment; (yellow) mock: K562 cells with Lipofectamine treated only (no RNA); (green) nonspecific RNA: nonspecific RNA control (AllStars negative control siRNA); (purple) 8-nt motif deleted: RNY5 sequence with nucleotides 14–21 motif deleted; (orange) 8-nt motif scrambled: RNY5 31-mer sequence with nucleotides 14–21 scrambled; (blue) RNY5 31-mer scram: 31-nt completely scrambled sequence; (red) DS RNY5: double stranded; (green) full-length RNY5 83-mer; (light green) RNY5 31-mer: 5′ RNY5 31-nt fragment.

Using EVs isolated from the BJ human primary cells, and four cancer (K562, HeLa, U2-OS, MCF7) cell lines, evaluations for the identification of phenotypic responses by cells taking up EVs were made. In each test, 2 × 10^5^ primary or cancer recipient cells were exposed to EVs obtained from approximately 10^8^ cells. This would result in a ratio of approximately 24,000 (BJ) or 500,000 (K562) EVs for each of the treated cells (Supplemental Table S1). Exposure of BJ cells to BJ EVs or K562 cells to K562 EVs (Fig. 4A,B) resulted in no observable cellular phenotype. However, exposure of primary BJ cells to EVs from each of the cancer cell lines resulted in a relatively rapid cell death phenotype (Fig. 4B).

To determine if the causative agent triggering this cell-death phenotype was the RNA cargo resident in the EVs, deproteinized and DNase-treated RNA was isolated from each of the EV preparations obtained from the BJ and K562 cell lines. The total RNA preparations from each of the cell lines were then transfected via lipofection into the BJ and K562 cell lines. Transfection of total RNA obtained from K562 EVs resulted in an approximately twofold increase (10.6% versus 20.5%) in the cell death of the BJ cells compared with BJ EV total RNA (Fig. 4B), while K562 cells were unaffected by the transfection of total K562 EV RNA (Fig. 4A).

Based on the notable abundance of the 31-nt processed product from the 5′ side of RNY5 in EVs, we investigated whether the cell-death phenotype was specifically attributable to this RNA. A total of four human primary (BJ, IMR90, HUVEC, HFFF) and four cancer (K562, HeLa, U2-Os, MCF7) cell lines were each transfected with a synthetic version of 31-nt processed *RNY5*. Each of the primary cells tested exhibited a cell-death phenotype while none of the cancer cell lines exhibited this phenotype (Fig. 4C). Varying the amounts of the synthetic 31-nt RNA resulted in a dose-dependent cell-death phenotype for BJ cells. (Fig. 4D)

Since other forms of *RNY5* can be detected in EVs, we decided to investigate if any of them may also contribute to the phenotype. Transfection of 23-nt oligonucleotide in BJ cells induced comparable levels of cell death to that seen with the 5′ 31-nt synthetic RNA (Fig. 4E). However, the 83-nt full-length *RNY5* RNA, the synthetic version of the 3′ 31-nt fragment, and a double-stranded version comprised of the 5′ and 3′ 31-nt species induced substantially lower levels of cell death in BJ cells (Fig. 4E). The levels of cell death triggered by these synthetic RNA products and observed in K562 cells were all similar and at background levels (Fig. 4F).

We hypothesized that the inability of double-stranded versions of the RNA to cause the phenotype may be related to sequestration of the 8-nt motif, the importance of which was demonstrated in the processing assays. This prompted us to investigate its role in causing the phenotype. We observed that the cell-death phenotype was lost when the motif was scrambled or deleted (Fig. 4E), further emphasizing the importance of this motif.

### Genome-wide gene responses associated with EV and processed *RNY5*

Comparison of transcriptional profiles prior to and at 24 h after treatment with EVs derived from K562 cells, as well as the synthetic version of the 31-nt form of *RNY5*, were made on two human primary cell lines (BJ and HUVEC). Of the 57,820 annotated genes in Gencode v19, we chose a twofold cutoff to characterize a gene as up- or down-regulated, which put >95% of the genes below this threshold and having a false discovery rate less than 0.05 (see Materials and Methods). In the case of BJ cells, 1945 annotated genes were seen to be differentially expressed greater than twofold 24 h after EV treatment, while treatment with the synthetic 31-nt oligonucleotide induced expression change in 1238 genes. Interestingly, 569 genes were observed to be commonly differentially expressed both after EV or oligonucleotide treatment. Similarly, 24 h after HUVEC cells were treated with EV or oligonucleotide, we observed 2493 genes and 1147 genes differentially expressed, respectively, of which 385 genes were commonly differentially expressed. The large number of genes commonly differentially expressed after EV treatment and 5′ 31-nt treatment in both BJ and HUVEC, suggests that the 31-nt RNY5 fragment by itself was able to recapitulate a significant part of the changes caused by EVs.

**FIGURE 5.**
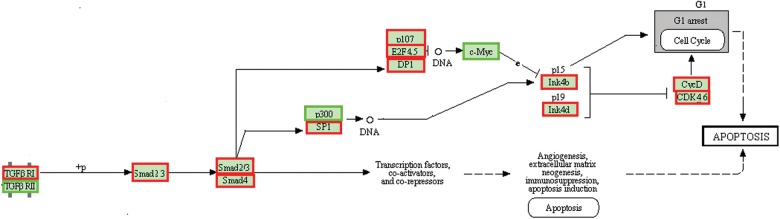
The part of the TGF-β pathway depicted from KEGG pathways, where we observe similar changes in transcript levels of genes between both types of treatments (K562 EV and the 32-nt synthetic RNA) in both BJ and HUVEC cells. The pathway highlights a common response of transcript levels of most genes in this part of the pathway.

Additionally, of the 1238 genes and 1147 genes that are differentially expressed after treatment with 5′ 31 nt in BJ and HUVEC cells, respectively, 141 genes are commonly differentially expressed. A gene set overrepresentation analysis for GO pathways with these commonly differentially expressed genes indicated significant enrichment of genes from pathways related to G2/M DNA replication checkpoints (*P*-value < 6.51 × 10^−3^), POU5F1 (OCT4), SOX2, NANOG activated genes related to proliferation (*P*-value < 1.72 × 10^−2^), activation of ATR in response to replication stress (*P*-value < 3.17 × 10^−2^), GRB7 events in ERBB2 signaling (*P*-value < 4.17 × 10^−2^). In agreement with previous studies regarding cancer EV-mediated cell death in primary immune cells (Taylor et al. 2003; Abusamra et al. 2005; Kim et al. 2005), we observed that transcriptional profiles of primary cells treated with EVs from cancer cells triggered differential expression of several genes associated with the *FAS/TGF-*β*-Smad2/3* apoptotic pathway. These same genes were significantly altered both by treatment with EVs or oligonucleotides in both primary cell types tested (GO process—signaling by TGF-β receptor activating *SMADs*—EV treatment [*P*-value < 4.4 × 10^−8^], RNY5 treatment [*P*-value < 8.8 × 10^−3^]) (Fig. 5). Also observable was the decrease in expression of the downstream *Ink 4b,* which is a negative regulator of *cyclin E*, *cyclinA*, and *CDK2*, and decreased expression of *SMAD2/3/4*, reinforcing the involvement of RNY5 in the cell cycle (Fig. 5). The absence of any potential cofactor accompanying the synthetic 31-nt RNA indicates that the RNA itself was sufficient to trigger the cell death phenotype (Supplemental Table S3; Fig. 4C).

### Evidence of primary cell targeting by cell to cell transfer

To determine whether selective primary cell death caused by cancer cells present in numbers that favored neither cell type, co-culture of cancer and primary cells at 1:1 ratio (i.e., 2 × 10^5^ cells for each cell type) were carried out. Co-culture conditions were of two types, first involving cell-to-cell contact and, second, separate growth of each cell type in permeable trans-well culture conditions. Approximately fourfold more cell deaths of primary cells (BJ) compared with untreated controls was observed in the cell-to-cell contact experiments (Supplemental Fig. S5). The results using a trans-well assay approach in which the primary and cancer cell populations were separated by ∼1 mm also demonstrated primary cell death, indicating that direct physical contact between cells and smaller volumes of media are not necessary for the occurrence of the phenotype.

## DISCUSSION

In this study, a short noncoding RNA as a component of EV cargo has been identified that can potentially play an important role in cancer cell microenvironments. Specifically, 31- and 23-nt processed fragments of *RNY5* have been identified as the most abundant and enriched RNA components of K562 and other cell type EVs. This processing of RNY5 into smaller fragments likely occurs within EVs and represents the first example, in our understanding, of EV RNA processing specifically for extracellular utilization. Most importantly, using a synthetic oligonucleotides-based approach, we report that ectopic overexpression of 31-nt processed fragments of RNY5 induce cell death in primary cells of multiple developmental origins in a dose-dependent manner, but fail to elicit a similar response from cancer cells. Furthermore, we show that the response is mirrored when BJ cells are treated with K562 EV RNA alone as well as with K562 EVs. Finally, an 8-nt motif in both 31- and a 23-nt RNY5 fragments has been identified as crucial for triggering cell death phenotype.

Single- and double-stranded RNAs are well-documented pathogen-associated molecular signals that are recognized by cytosolic receptors of the innate immune system of many cell types during virus infection (Saito and Gale 2008). This recognition of exogenous RNAs can result in the activation of caspase-1 and subsequent apoptosis of affected cells (Mogensen 2009). Differentiation of endogenous from exogenous RNAs is partially based on the presence of 5′ triphosphate or poly-uracil or -adenylyl strings frequently found in RNA viral genomes (Takeuchi and Akira 2009). The single-stranded *RNY5* 31- and 23-nt processed sRNAs lack these viral signals and are compartmentalized within vesicles. Interestingly, a double-stranded version of the 31-nt processed product triggers a substantially lower cell death phenotype, unlike that seen with the antiviral innate immune responses. The 83-nt primary h*Y5* transcript, which is reported to form a very stable hairpin structure (van Gelder et al. 1994; Maraia et al. 1996) and thus likely renders the 23- and 31-nt regions inaccessible, also triggers substantially lower cell deaths. Interestingly, Gardiner et al. (2009) and Wang et al. (2014) reported a double-stranded version of the critical 8 nt found in the RNY5 sRNA (5′GUAGUGGG3′) to be sufficient for RNY1 to support the initiation of DNA replication. However, in our studies it is clear that the *hY*5 31- and 23-nt processed products triggered cell death is attenuated in the presence of their complementary strands. Although Gardiner et al. (2009) did not report if a single-stranded version of this sequence was capable of supporting the initiation of replication, one possibility is that the single-stranded 31- and 23-nt sRNAs cause inappropriate and perhaps uncontrolled DNA replication signals in primary cells, triggering cell death. Such processed *RNY5*-stimulated signals might be less effective in cancer cell lines given their characteristic loss of DNA replication controls inherent with transformed cells.

Two sets of results reported in these studies prompt testable hypotheses concerning the biological and mechanistic outcomes that may be observed in the follow-up in vivo studies. Although cell death of primary cells is readily detected and this cell death is observed to be related to the dose of 5′ *RNY5* 31-nt fragment, it is notable that not all exposed primary cells die. Different proportions of primary cells survive depending on the primary cell type and dosage used. These results appear to indicate that not all cultured cells are equally sensitive. Recent reports indicate that tumor-fibroblast interactions act in parallel to promote tumorigenicity and not all associated primary fibroblast cells may be involved in this cooperational activity (Rajaram et al. 2013). One testable hypothesis in future studies is to determine whether the surviving primary cells after treatment with either cancer cell EVs or the 31-nt processed product continue to fail to respond to the exposure of the 31 nt or EVs or if they do provide support for tumor growth.

A second set of results that potentially could help to focus future in vivo experiments concern the observation that although the 31- and 23-nt sRNAs are present in the EVs from both primary and cancer cells, exposure of EVs isolated from BJ cells do not trigger cell death in BJ cells. One possibility consistent with these results is that different cofactors present in primary and cancer cell EVs and are associated with the 31- or 23-nt cargos depending on their origin. Increased quantities of EVs released by cancer cells and relative abundance of processed RNY5 transcripts in cancer cell derived EVs may also contribute to this differential response.

Detailed understanding of the molecular mechanisms involved in 31-nt RNY5 induced cell death would be crucial in understanding the differential response by primary and cancer cells. Investigations are currently underway to elucidate the type of cell death induced by 31-nt RNY5. These results also prompt us to investigate the protein binding partners of RNY5 in cancer and primary cells as well as in their respective EVs. Identification of RNY5-binding proteins will not only provide us mechanistic insights of the observed response, but may also reveal the molecular mechanisms involved in specific sorting and processing of RNY5 transcripts into EVs.

In the late 19th century, Paget proposed the “seed and soil” hypothesis indicating that the microenvironment (soil) was key for tumor (seed) growth (Paget 1989). Increasingly, the importance of the tumor microenvironment has been recognized as a key contributor for cancer progression and drug resistance (Kaplan et al. 2006; Bidard et al. 2008; Mendoza and Khanna 2009; Peinado et al. 2011; Heinrich et al. 2012). It has been hypothesized that a component for establishing and maintaining supportive microenvironments are the contents of EVs (Hood et al. 2011). Uncovering the functional role of processed RNY5 transcripts orchestrated through extracellular vesicles reveals an intricate competitive cell interaction mechanism, potentially involved in promoting the establishment of a microenvironment for the spread of tumor cells. While further studies are warranted to evaluate a possible in vivo role for the RNY5 fragments in the tumor microenvironment, it raises an interesting possibility that RNY5 fragment-induced cell damage and lethality may also sensitize normal tissue to neoplastic cell invasion and metastasis by promoting cell removal and inducing inflammatory response.

## MATERIALS AND METHODS

### Isolation of extracellular vesicles (EVs)

K562 cells were grown in complete RPMI1640 medium (10% FBS + 1% penicillin-streptomycin), and BJ cells were grown in DMEM (10% FBS and 1% penicillin-streptomycin). When the cells reached approximately 70%–80% confluence, the media were replaced with serum-free conditioned medium and incubated for another 24 h. The conditioned medium was then centrifuged at 300*g* for 10 min. The cell pellet was discarded and the supernatant was further centrifuged at 2000*g* for 10 min. The pellet comprised of mostly cell debris and apoptotic bodies was discarded and the supernatant was again centrifuged at 10000*g* for 30 min. The pellet comprised of microvesicles was discarded and the supernatant was filtered at 3500*g* for 15 min using Centricon Plus70 100KD NMWL cut-off (Millipore). The filtrate was discarded and the residue, enriched with EVs and other proteins, was collected. The collected residues were precipitated overnight using ExoQuick-TC (System Biosciences) at 1:5 ratio (by volume) of Exo-quick to filtration residue. The next morning, the sample was centrifuged at 1500*g* for 30 min. The supernatant was discarded and the pellet was centrifuged again at 1500*g* for 5 min. Leftover supernatant, if any, was discarded and the pellet was resuspended in 500 μL PBS.

### Electron microscopy

Negative staining of EV suspensions followed by imaging in a transmission electron microscope was used to determine vesicle shape and size distribution (Raposo et al. 1996). Aliquots of EV suspensions were dispensed onto sheets of Parafilm in a humidified petri dish and the vesicles were adsorbed onto freshly prepared Butvar coated EM grids (glow discharged). The adsorption was done for 5 min at room temperature. The petri dish containing the suspensions and EM grids was transferred to a large bucket of ice shavings and the grids were transferred to three successive drops of distilled water (30 sec each) to remove salts, then transferred to a drop of 1% uranyl acetate in 1% methyl cellulose for 30 sec, and then placed in a second drop of negative stain solution for 5 min. Excess stain was blotted off and the grids were air dried.

Immuno-gold labeling for the CD81 was done by resuspending the EVs in primary mouse antibody to human CD81 (Abcam) diluted in PBS for 30 min at room temperature. Incubations were done in sterile 1.5 mL micro centrifuge tubes. The antibody labeled vesicles were pelleted by centrifugation, resuspended in a 1:10 solution of 5 nm colloidal gold conjugated to rabbit antimouse IgM secondary antibody (Aurion, Electron Microscopy Sciences) for 30 min. The gold labeled vesicles were adsorbed to Butvar-coated grids for 5 min and then rinsed through 3 drops of PBS to remove unbound gold particles. Negative staining of the gold labeled vesicles was completed as described above.

Samples were imaged in the Hitachi H7000 Electron Microscope operated at 75 kV. Images recorded on Kodak EM film 4489 were scanned at 2400 DPI on an Epson Perfection V750 film scanner.

### Western blot

Proteins were isolated using RIPA buffer (Pierce) following the manufacturer’s protocol, concentrated using Amicon Ultra 3K centrifugal filter (Millipore), and quantified using the BCA Protein Quantification kit (Pierce). One microgram of protein from K562 whole cell and EVs was loaded on precast 4%–20% Tris-Glycine gel (Life Technologies) and transferred to PVDF membrane. The membrane was blocked using Pierce TBST blocking buffer (cat. no. 37571) for 1 h at room temperature. Primary antibody incubation was performed overnight at 4° at 1:1000 dilutions, while secondary antibodies were used at 1:10000 dilutions. Membranes were developed with Amersham ECL plus Western Blotting Development kit (GE). Anti-fibrillarin (Abcam, cat. no. ab18380), anti-protein disulphide isomerase (Abcam, cat. no. ab2792), and anti-prohibitin (Abcam, cat. no. ab28172) were used as nuclear, endoplasmic reticulum, and mitochondrial marker, respectively. Anti-PDC6I (Abcam, cat. no. ab88743), anti-Tsg101 (Abcam, cat. no. ab83) and anti-transferrin receptor (Abcam, cat. no. ab84036) were used as EV markers. Goat polyclonal to rabbit IgG (Abcam, cat. no. ab6721) and rabbit polyclonal to mouse IgG (ab 6728) were used as secondary antibodies.

### NTA

Quantification of the extracellular vesicles was performed by Nanoparticle Tracking Analysis (NTA) using NanoSight LM10 at 25°C. PBS was used as a diluent and samples were run at 1:500 dilutions for K562 EVs and 1:5 dilutions for BJ EVs.

### Isolation of RNA

RNA isolation was performed using Ambion's Mirvana miRNA Isolation kit (cat. no. AM1560) following the manufacturer's protocol. Prior to RNA isolation, EVs were treated with Ambion RNase cocktail (cat. no. AM2286) at 37° for 15 min. One milliliter of lysis/binding buffer was immediately added to the RNase treated EVs to deactivate the RNase.

### Detergent and RNase treatment

To determine if the isolated RNA was true EV RNA cargo and not an artifact of purification, RNA isolate from EVs without RNase treatment was compared with RNA isolate from RNase treated EVs and RNA isolate from detergent and RNase treated EVs. RNase treatment of EVs resuspended in PBS was performed with Ambion RNase cocktail at 37° for 15 min. Detergent treatment was performed with RIPA buffer for 15 min followed by RNase treatment as described above.

### Small RNA sequencing

Small RNA was isolated with Mirvana miRNA Isolation kit (Life Tech) and DNase treated with Ambion Turbo-DNase (Life Tech). Ribosomal RNA depletion was performed on whole cell RNA using the Eukaryote Ribominus kit (Life Tech) following the manufacturer's protocol. Both EV and whole cell RNA were treated with tobacco acid pyro-phosphatase (Epicenter) to make 5′ capped and triphosphate RNAs amenable to adapter ligation. Libraries were constructed using Illumina TruSeq Small RNA kit according to the manufacturer’s protocol, except reverse transcription was performed using Superscript III. Amplified libraries were run on 2% agarose gel and the 20–200 nucleotide (nt) region was cut and gel-purified with Qiagen Gel Extraction kit. Libraries were quantified on Agilent Bio-analyzer HS-DNA chip and sequenced on Illumina HiSeq2000.

### Long RNA sequencing

Long RNA was isolated with Mirvana miRNA Isolation kit and DNase treated (Ambion, cat. no. AM2238) following the manufacturer’s protocols. Construction of complementary-DNA libraries was performed using Illumina TruSeq Stranded Total RNA kit (cat. no. RS-122-2201). Libraries were quantified using Agilent Bioanalyzer HS-DNA chip and run on Illumina Hi-Seq 2000 platform.

### Bioinformatics analyses

All data from RNA sequencing experiments in the study were mapped to Human Genome version 19 (hg19, GRCh37) obtained from the UCSC Genome Browser website (http://hgdownload.cse.ucsc.edu/downloads.html). RNA-seq reads were aligned using the STAR v1.9 software, and up to five mismatches per alignment were allowed. Only alignments for reads mapping to 10 or fewer loci were reported. Annotations were not utilized for mapping the data. The obtained BAM files were further processed using HTSeq software in order to appropriate the number of reads originating from each annotated region of the genome, utilizing annotations obtained from Gencode v19 of the human genome; using the “Union mode” option of the software for all libraries, tRNA annotations were obtained from tRNAscan database (Schattner et al. 2005). Reads per million (rpm) values for each gene were obtained by dividing the number of reads uniquely mapping within the limits of a gene annotation by the total number of uniquely mapping reads in the library and multiplying by a million. These rpm values were used between replicates in Supplemental Figures S2A,B to establish correlation between biological replicates of EV RNA libraries. Relative abundance of RNA families in Figures 2A,B,C,D was calculated using the cumulative rpm values of all genes within the Gencode defined RNA families (such as miRNA, snoRNA, miscellaneous RNA [miscRNA], protein coding etc.). Within each pie chart in Figure 2, the group termed as “Others” includes all categories of Gencode other than lincRNA, miRNA, miscRNA, rRNA, tRNA, snRNA, snoRNA, and protein coding genes (such as 3prime_overlapping_ncrna, immune-globulin genes, mitochondrial tRNA, mitochondrial rRNA, anti-sense RNA, antisense, pseudogenes, T-cell receptor genes, sense_intronic, sense-overlapping genes etc.). Density plots in Supplemental Figures 3A,B were obtained by calculating the ratio of rpm within EV to the sum of rpms within EV and whole cell for both K562 and BJ cells. The density function for genes of each RNA family within these graphs was calculated from these ratios using the kernel density function within the R stats package.

Fragment analysis to identify the most commonly found fragments within the RNY5 gene was found by taking into account start and end positions of all reads that mapped to the RNY5 gene from chromosome 7 between positions 148638580 and 148638658 in the positive strand. All reads which began at the 5′ end of the RNY5 gene and >29 nt in length mapped uniquely to the RNY5 gene. Similarly, reads that began in places other than the 5′ end of the RNY5 gene mapped uniquely to the gene’s primary location on chromosome 7. However, genes which started in the 5′ end of the gene and were 29 nt in length or shorter were all multimappers and mapped with 100% identity to two other locations (chromosome 12:45581224–45581252 and chromosome 13:103472349–103472369) and 97% identity to a few other locations (chromosome 12:98223788–98223816, chromosome 19:36540048-36540076, and chromosome 1:35893466–35893493), thus making it impossible to accurately establish the true origin of these reads absolutely. These locations are annotated as pseudogenes of the RNY5 gene, and to resolve this uncertainty of their origin we included them for the fragment analysis. The secondary structure of RNY5 was obtained using the online resource of the Mfold package, within which the most frequently occurring fragments were highlighted.

In order to identify genes which are differentially expressed (DE) between time points for the molecular phenotype section, bio-replicates from 24 h after treatment with EVs were compared to the untreated replicates, by using DESeq on the read counts of the genes derived from the HTSeq software, filtering by false discovery rate (FDR) less than or equal to 0.05 and by fold-change greater than or equal to 2 or less than or equal to 0.5. The list of DE genes common to the two cell types on treatment with K562 EVs and the list of DE genes common to the two cell types after 5′ 32mer treatment were then used for further overrepresentation analysis on the GO biological processes using the online resource of Reactome Pathways (http://www.reactome.org), where only biological processes with a *P*-value <0.05 were taken to be significant. The list and map of genes within the FAS/TGF-β pathway were obtained from KEGG pathways, and those genes within DE gene lists were overlaid on the map, where red color indicates a fold change below 0.05, green indicates fold change greater than 2, and blue indicates no significant fold change after treatment in each cell type.

### Lipid labeling of EVs and imaging

K562 EVs were isolated as described above. Two microliters of PKH67 (Sigma, cat. no. MINI67-1KT) were resuspended in 500 µL diluent and added to purified EVs for 4 min in the dark, and EVs were isolated using Exoquick-TC as described above. The labeled EV pellet was resuspended in complete medium (DMEM + 10%, FBS + 1% penicillin-streptomycin) and added to BJ cells for overnight incubation. Imaging was done on Deltavision OMX microscope and image analysis was performed with Delta-vision SoftWorx software.

### Metabolic labeling of RNA and imaging

K562 cells (2 × 10^7^) were incubated at a final concentration of 0.2 milliMolar 5-ethynyl uridine (EU) for 24 h. EVs were isolated from the conditioned medium as described above. 3T3 cells were treated with actinomycin D at a final concentration of 1 μM for 1 h to block its endogenous transcription. The drug-treated media were replaced with fresh complete DMEM medium and the cells were incubated with EU labeled K562 EVs for 2 h. The cells were subsequently fixed with 4% para-formaldehyde and permeabilized with 0.5% Triton-X-100. EU incorporated EV RNA was detected using Click chemistry according to the manufacturer's protocol (Life Tech, cat. no. C10329) and nuclei were counterstained using Hoechst 33342. Finally, cells were imaged on Delta-vision OMX microscope and image analysis was performed with Delta-vision SoftWorx. As a negative control, 3T3 cells treated with actinomycin D and directly incubated with EU was performed which showed no signal of EU-incorporated RNA, thus confirming block of endogenous transcription (data not shown).

### Subcellular localization of RNY5 31-mer

A total of 2 × 10^5^ BJ cells were plated overnight and next morning cells were transfected with 100 picomoles of synthetic RNY5-31mer coupled with Alexa 488 fluorophore at its 3′ end. After 6 h, transfection medium (Opti-MEM) was replaced with complete DMEM medium and incubated for another 24 h. Imaging was performed on Delta-Vision OMX microscope and Image processing was performed with Delta-vision SoftWorx software.

### Interspecies transfer of RNA by RNA-seq

Mouse HB4 cells (ATCC) were treated with K562 EVs for 0, 12, and 24 h, HB4 cells were untreated (neg. control), and RNA isolation was performed using Mirvana miRNA Isolation kit. Isolated RNA was ethanol-precipitated, DNase treated, and size separated into long (>200 nt) and short RNA (<200 nt). The short RNA was ribo-depleted using Ribo-minus Eukaryote Ribo-depletion kit (Life Tech) following the manufacturer's protocol, and ethanol precipitated. The precipitated RNA was then treated with Tobacco Acid Pyro phosphatase (Epicenter) at 37° for 1 h to convert the 5′ capped and triphosphate RNA molecules into 5′ monophosphate and make them amenable for adapter ligation. RNA was then purified by phenol-chloroform treatment followed by ethanol precipitation. The small RNA libraries were then constructed using A-tailing protocol as described in Djebali et al. (2012). The amplified libraries were then run on 2% agarose gel and the region between 20–200 nt was cut and gel extracted with Qiagen Gel Extraction kit. Finally, libraries were quantified using Agilent Bioanalyzer and sequenced on Illumina MiSeq platform. Mapping was performed by STAR against combined human and mouse genome and reads that mapped uniquely to humans only were considered for analysis. RNY5, a human specific gene enriched in EVs, was used as a marker to demonstrate interspecies transfer of human K562 EV RNA to mouse HB4 cells.

### Oligonucleotide end-labeling

Oligonucleotides (90 pmol for DNA oligonucleotides and 15 pmol for RNA oligonucleotides) were end-labeled in reactions containing 20 μCi of γ-^32^P-ATP (PerkinElmer), 5 units T4 polynucleotide kinase (New England BioLabs), 70 mM tris-HCl pH 7.6, 10 mM MgCl_2_, and 5 mM dithiothreitol (DTT). Labeling proceeded for 30 min at 37°, followed by phenol-chloroform extraction.

### Northern blots

Whole cell total RNA and EV RNA from K562 and BJ cells (850 ng each) were separated on 8% acrylamide, 8 M urea gels. Thereafter, the RNA was blotted to nitrocellulose membranes (Zeta-Probe, Bio-Rad). The blots were probed with an oligonucleotide complementary to the 5′ end of the RNY5 transcript (5′-CTTAACAATAACCCACAACACTCGGACCAACT-3′).

### In vitro processing

K562 whole cell and EV proteins were extracted with RIPA buffer (Thermo Scientific). Cold processing reactions contained the indicated amount of protein, 10 mM MgCl_2_, 10 mM DTT, and 2 pmol synthetic full length RNY5 RNA where indicated. After 30 min incubation at 37°, reactions were phenol-chloroform extracted, separated on 8% acrylamide, 8 M urea gels, then blotted and probed as described for Northern blots. Hot processing reactions were performed with synthetic versions of wild type RNY5 5′ 31-mer, shuffled 31-mer (5′-UGGUGCGUGUUGUUUAGAUUAAGUGGUUGAC-3′) or RNY5 31-mer with the 8 nt motif shuffled (GUUGUGGG→ACGUACAG). Each reaction contained 4 μg of K562 EV protein extract where indicated, 10 mM MgCl_2_, and 0.15 pmol of end labeled RNA. After 2 h incubation at 37°, samples were separated on 8% acrylamide, 8 M urea gels. Thereafter, the gels were subjected to autoradiography.

### RNA transfection

A total of 2 × 10^5^ cells were plated in 6-well plates overnight. The next day, RNA transfection was performed with Lipofectamine 2000 and Opti-MEM medium for 6 h according to the manufacturer's protocol. After 6 h, Opti-MEM media were replaced with complete medium and cells were incubated for another 24 h.

### Flow cytometry

Quantification of cell death was performed on a BD LSR-II Cell Analyzer (BD Biosciences) using a flow cytometry kit that detects membrane permeability, chromatin condensation, and dead cell apoptosis (Life Tech, cat. no. V23201). YO-PRO-1 was excited by the 488 nm laser and its emission was collected with a 530/30 filter. A 405 nm Violet laser was used to excite Hoechst 33342 and emission was collected with a 440/40 filter. Unstained cells and single color control samples (YO-PRO-1 only and Hoechst33342 only) were used for setting the PMT voltages and eliminating any spectral overlap between these two fluorochromes. Only events positively labeled with Hoechst33342 were considered for quantification. Cells double-labeled with Hoechst33342 and Yo-Pro-1 were quantified as “dead cells”; cells labeled with Hoechst33342 but not with Yo-Pro-1 were quantified as “living cells.” YO-PRO1, a nucleic acid binding dye, which is permeable to apoptotic and dead cells but not living cells, was used for quantification of cell death. Briefly, cells were trypsinized and resuspended in 800 μL DMEM medium. Cells were labeled with 1 μL of YO-PRO1 and Hoechst 33342 for 15 min at room temperature. The labeled cells were kept on ice and then passed through a cell strainer prior to running on the LSR-II.

### EV incubation with cells and cell death quantification

EVs were isolated from 1 × 108 cancer (K562, HeLa, U2-Os, and MCF7) or primary (BJ) cells as explained above and incubated with BJ or K562 cells for 24 h. After 24 h, quantification of cell death was performed by flow cytometry as explained above.

### EV RNA transfection and quantification of cell death

EV RNA was isolated from K562 and BJ EVs in duplicates with Mirvana miRNA Isolation kit as explained above. RNA transfection was performed with Lipofectamine 2000 and cell death quantification was performed after 24 h incubation by flow cytometry as described above.

### Synthetic ribonucleotide transfection and cell death quantification

A total of 2 × 10^5^ BJ or K562 cells were plated overnight, and the next day cells were transfected with 100 pmol of RNY5 31-mer and 100 pmol RNY5 23-mer with Lipofectamine 2000 in Opti-MEM medium. After 6 h, Opti-MEM media were replaced with complete DMEM media (for BJ) or complete RPMI1640 medium (for K562). Untreated and mock treatment were used as negative controls. AllStars negative control siRNA was used as nonspecific RNA control. A 31 nt scrambled RNA oligo was used as a scrambled RNA control. Furthermore, RNA oligonucleotides with 8 nt motif (nucleotides 14–21) scrambled, scrambled with secondary structure intact and 8 nt motif deleted oligonucleotide were used as controls for identifying the motif sequence responsible for the phenotype. Finally, transfection of 83 nt full length RNY5 and a double stranded RNY5 31-mer shows substantially lower cell death.

### Generality of the phenotype

Generality of RNY5 31-mer mediated cell death phenotype was assessed in four cancer cells (K562, HeLa, U2-Os, MCF7) and four primary cells (BJ, HUVEC, IMR90, human fetal foreskin fibroblast [HFFF]). In each case, 2 × 10^5^ cells were plated overnight. The next day, cells were transfected with100 pmol of synthetic RNY5 31-mer (except HFFF, which was transfected with 200 pmol of RNY5) and Lipofectamine 2000 as described above. Cell death quantification was performed after 24 h incubation as described above.

### Dose response curve of RNY5-31mer

Transfection of BJ cells was performed with RNY5 31-mer and Qiagen AllStars negative control siRNA (nonspecific RNA control) in a dose dependent manner. Briefly, 2 × 10^5^ cells were plated overnight, and on the following day cells were transfected with RNY5-31mer (10, 50,100, 200, 300, and 400 pmol) or AllStars control (10, 50,100, 200, 300, and 400 pmol) with 10 μL Lipofectamine in Opti-MEM medium. Both untreated and mock treated (Lipofectamine only) was also performed as negative controls. After 6 h, media were replaced with complete DMEM medium and incubated for another 24 h. Quantification of cell death was performed as described above.

### Co-culture and cell death quantification

Co-culture of K562 and BJ cells was performed as direct co-culture as well as transwell co-culture. In the direct co-culture system, 2 × 10^5^ BJ cells were plated on 6-well plates, and the next day cells were labeled with Hoechst33342 for 15 min in the dark at 37°. Cells were washed thrice with PBS and replaced with complete DMEM medium. 2 × 10^5^ K562 cells resuspended in 2 ml RPMI1640 medium were added to the same well and directly co-cultured with BJ cells. As a negative control, BJ cells were grown alone in 2 ml DMEM + 2 ml RPMI1640 medium. After 24 h, both cells were harvested together but were only labeled with YO-PRO-1. Quantification of cell death was performed by flow cytometry as described above. Since K562 cells, although present in the solution were not labeled with Hoechst 33342, Hoechst33342 and YO-PRO-1 double labeled cells were quantified as “dead BJ cells” while Hoechst 33342 positive and YO-PRO-1 negative cells were quantified as “living BJ cells.”

In the Transwell co-culture system, 2 × 10^5^ BJ cells were plated at the bottom of the well. The next day, 2 × 10^5^ K562 cells were plated in RPMI medium in the same well but across a Transwell membrane (Corning, 1 μm pore size). After 24 h, K562 cells on top of the membrane were discarded while the BJ cells on the well were labeled with YO-PRO-1 and Hoechst33342, and flow cytometry was performed for quantification as described above.

### Synthetic RNA oligonucleotides sequences

RNY5 31-mer: 5′-rArGrU rUrGrGrUrCrCrGrArGrUrGrUrUrGrUrGrGrGrUrUrArUrUrGrUrUrArA-3′

RNY5 23-mer: 5′-rArGrUrUrGrGrUrCrCrGrArGrUrGrUrUrGrUrGrGrGrUrU-3′

RNY5 31nt complete scrambled: 5′-rUrGrGrUrGrCrGrUrGrUrUrGrUrUrUrArGrArUrUrArArGrUrGrGrUrUrGrArC-3′

RNY5 8 nt motif deleted: 5′-rArGrUrUrGrGrUrCrCrGrArGrUrUrUrArUrUrGrUrUrArA-3′

RNY5 31-mer with 8 nt motif scrambled: 5′-rArGrUrUrGrGrUrCrCrGrArGrUrArCrGrUrArCrArGrUrUrArUrUrGrUrUrArA-3′

RNY5 32-mer complementary (3′ side) fragment: 5′-rCrCrCrCrArCrArArCrCrGrCrGrCrUrUrGrA rCrUrArGrCrUrUrGrCrUrGrUrUrU-3′

Full length RNY5 83-mer: 5′-rArGrUrUrGrGrUrCrCrGrArGrUrGrUrUrGrUrGrGrGrUrUrArUrUrGrUrUrArArGrUrUrGrArUrUrUrArArCrArUrUrGrUrCrUrCrCrCrCrCrCrArCrArArCrCrGrCrGrCrUrUrGrArCrUrArGrCrUrUrGrCrUrGrUrUrU-3′

Double-stranded RNY5 31-mer: 5′-rArGrUrUrGrGrUrCrCrGrArGrUrGrUrUrGrUrGrGrGrUrUrArUrUrGrUrUrArArG-3′

5′-rCrCrCrCrArCrArArCrCrGrCrGrCrUrUrGrArCrUrArGrCrUrUrGr CrUrGrUrUrU-3′

## SUPPLEMENTAL MATERIAL

Supplemental material is available for this article.

## Supplementary Material

Supplemental Material
