# Supplementary material for: Extracellular vesicle-mediated transfer of processed and functional RNY5 RNA
Source: RNA. 2015 Nov;21(11):1966–79. doi: 10.1261/rna.053629.115 (PMC4604435; doi:10.1261/rna.053629.115)
Supplement: Supplemental Material [file supp_21_11_1966__index.html]

Extracellular vesicle-mediated transfer of processed and functional RNY5 RNA — Supplemental Material 

# Extracellular vesicle-mediated transfer of processed and functional RNY5 RNA

## Supplemental Material

**Files in this Data Supplement:**

- Supp Figure S1.pdf
- Supp Figure S2.pdf
- Supp Figure S3.pdf
- Supp Figure S4.pdf
- Supp Figure S5.pdf
- Supp Table S1.pdf
- Supp Table S2.pdf
- Supp Table S3.pdf
- Supp Table S4.pdf
- Supp Legends.docx
