# Supplementary figures and images for: Extracellular vesicle-mediated transfer of processed and functional RNY5 RNA
Source: RNA. 2015 Nov;21(11):1966–79. doi: 10.1261/rna.053629.115 (PMC4604435; doi:10.1261/rna.053629.115)

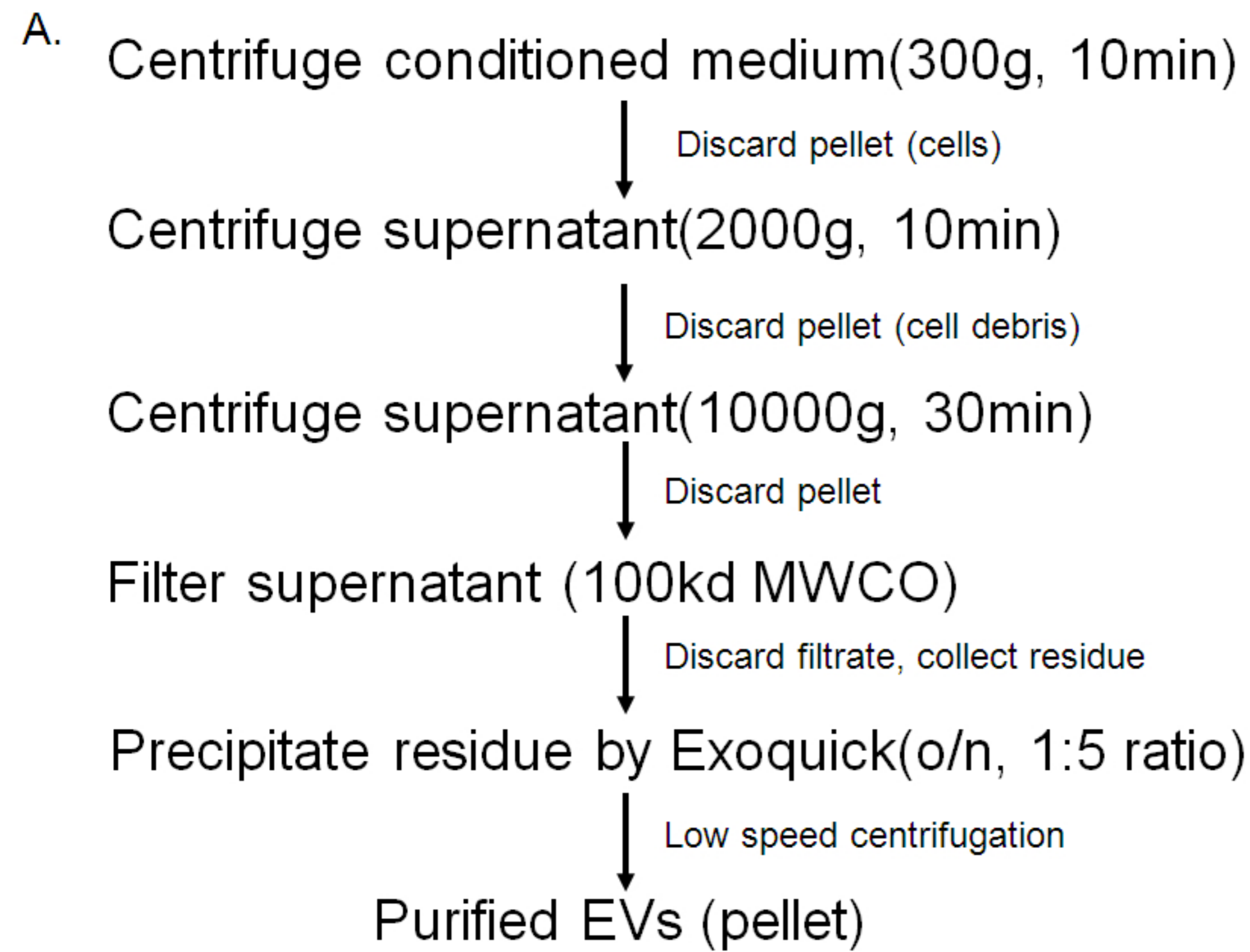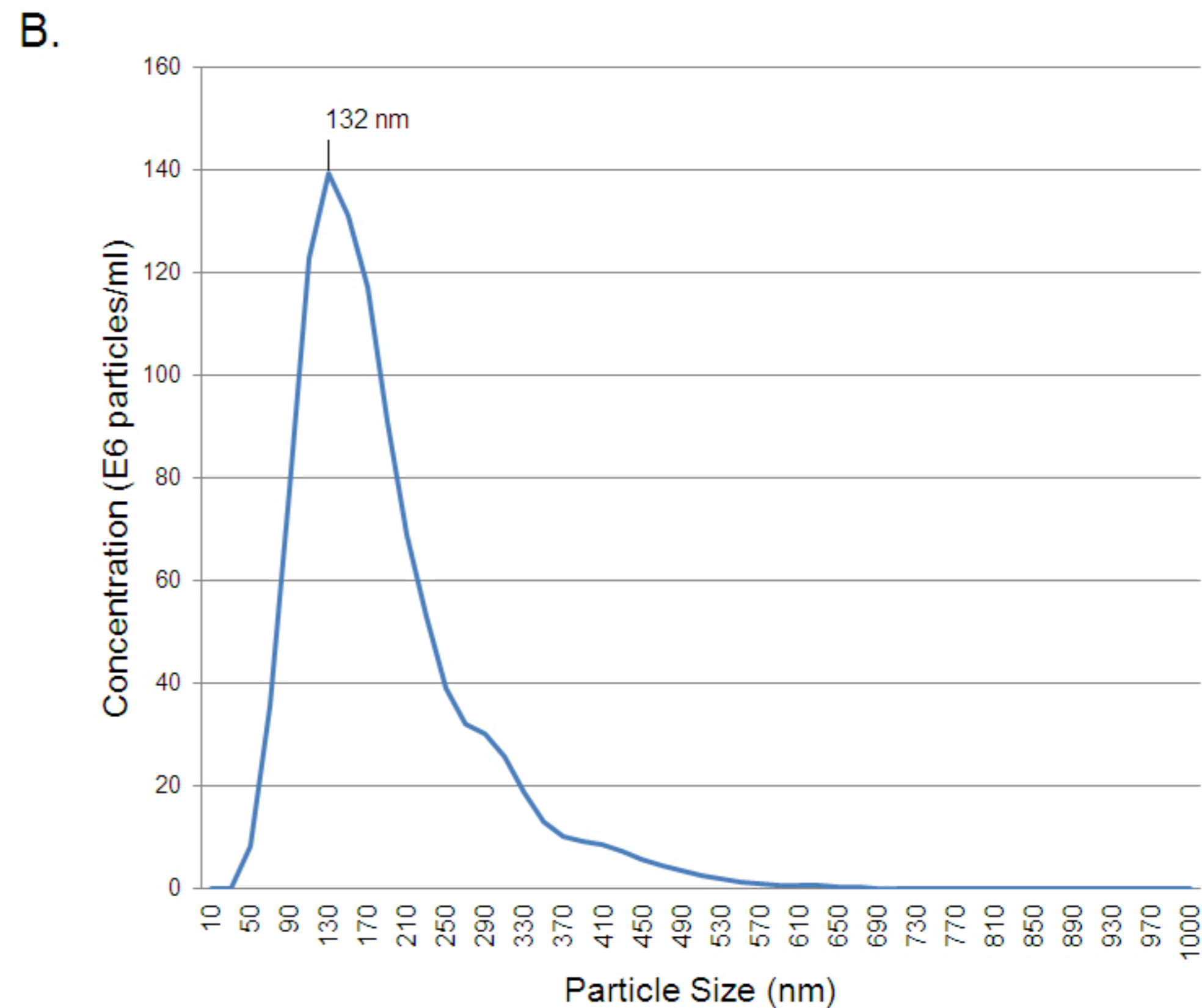

Supplement: Supplemental Material [file supp_053629.115_FigureS1.pdf]

A.

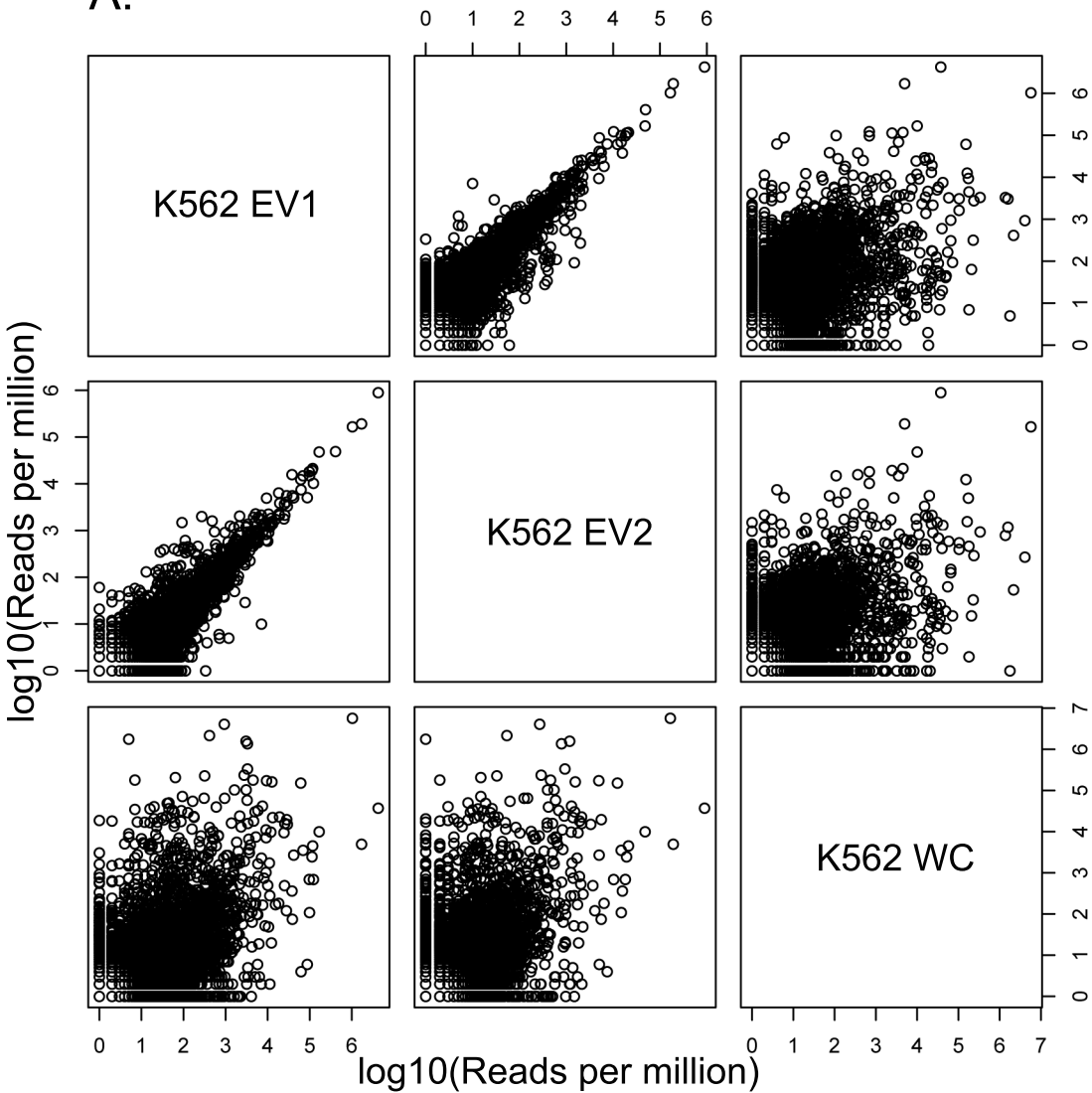

B.

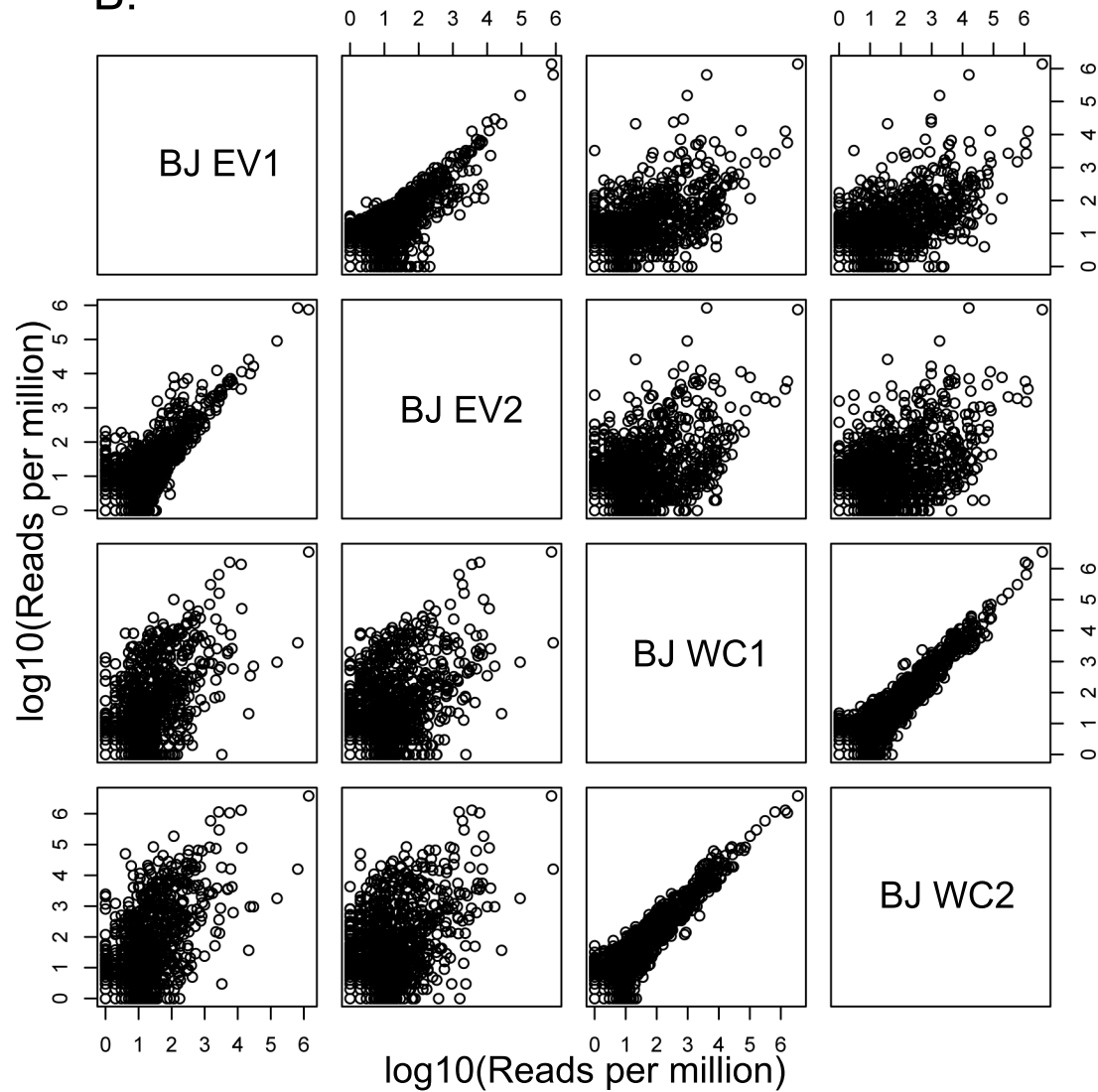

Supplement: Supplemental Material [file supp_053629.115_FigureS2.pdf]

A.

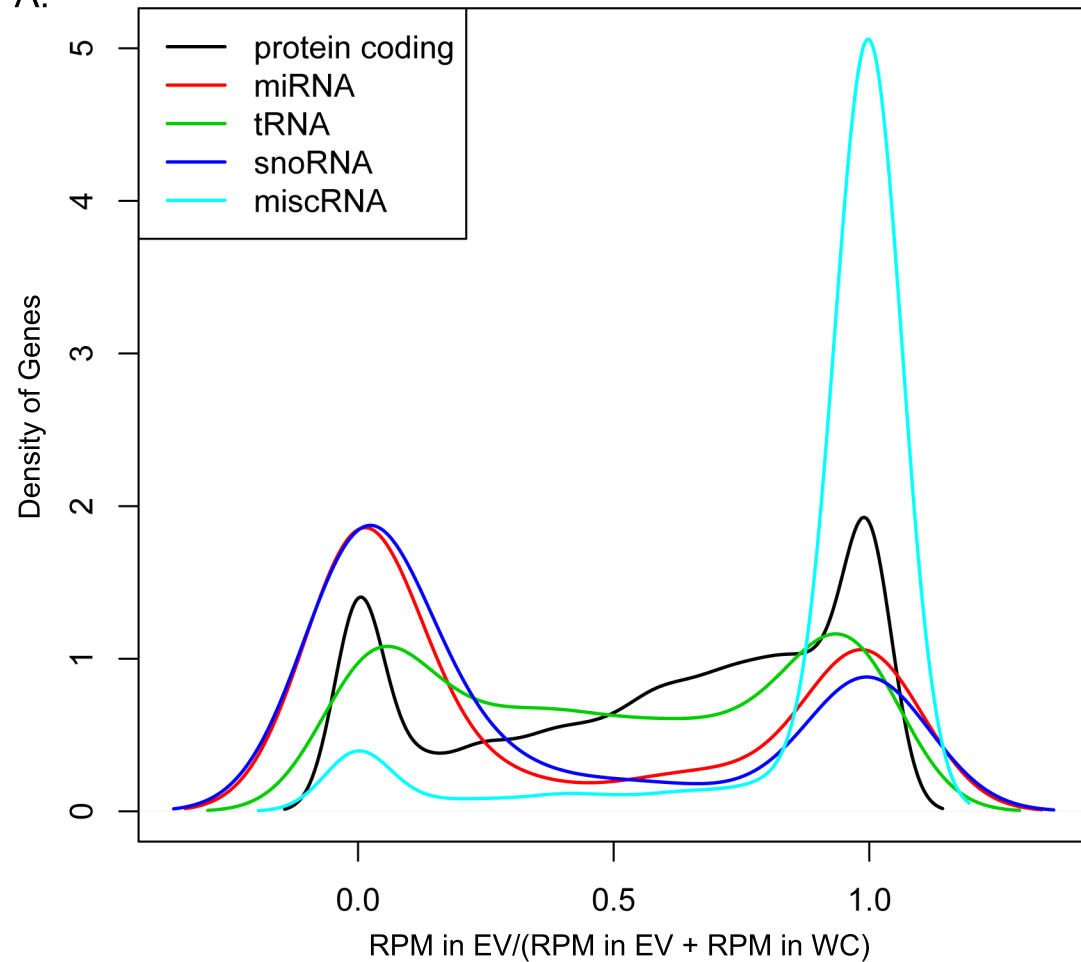

B.

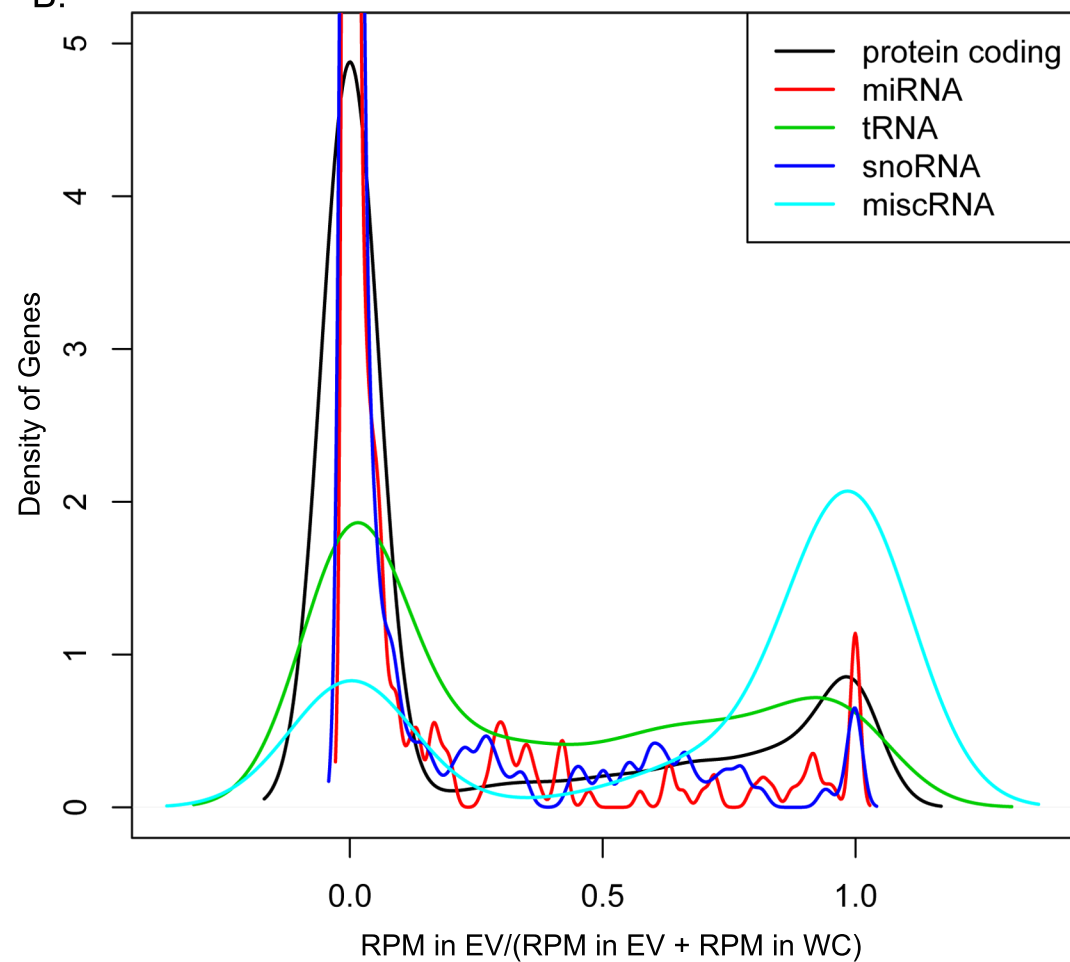

Supplement: Supplemental Material [file supp_053629.115_FigureS3.pdf]

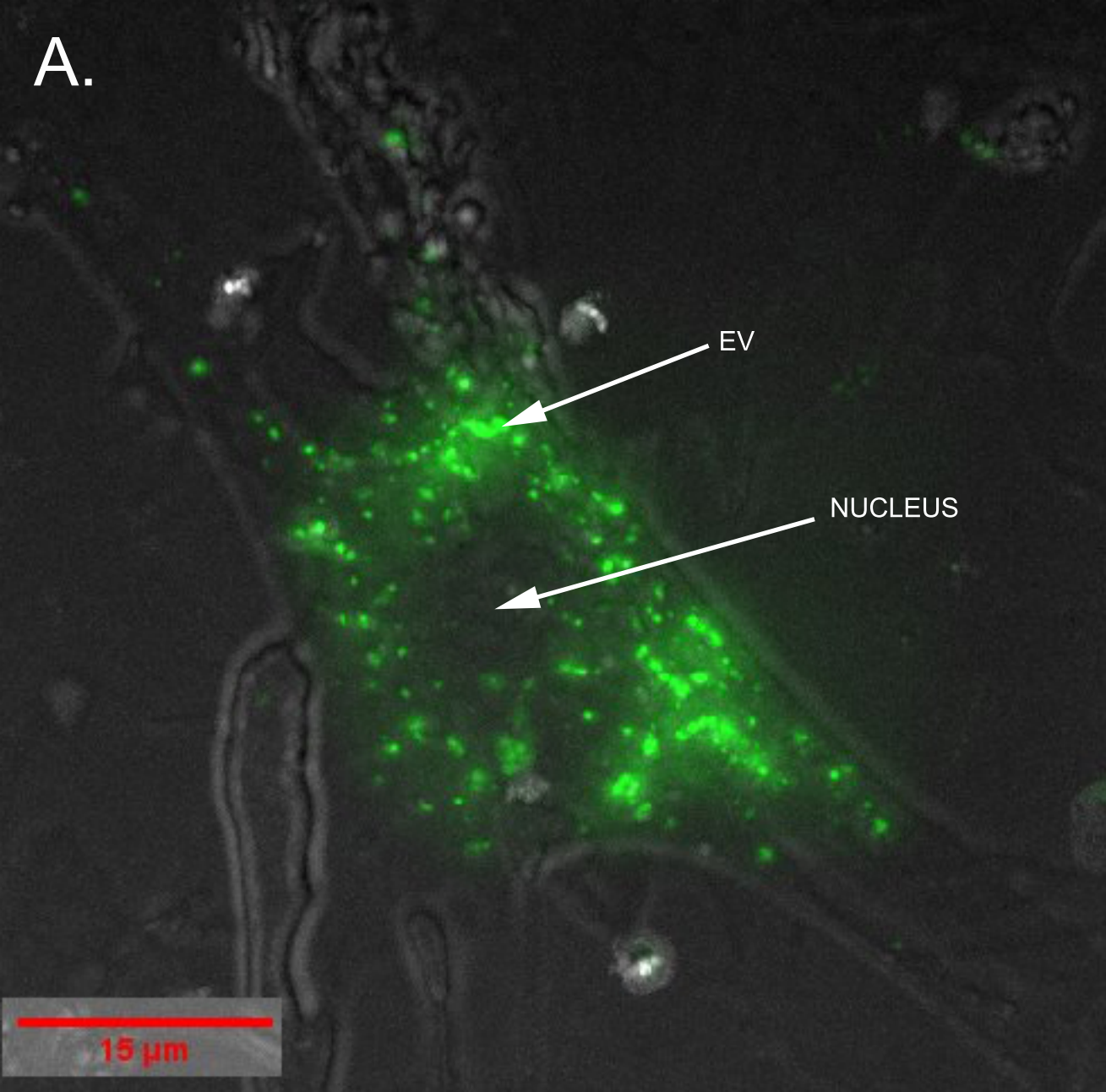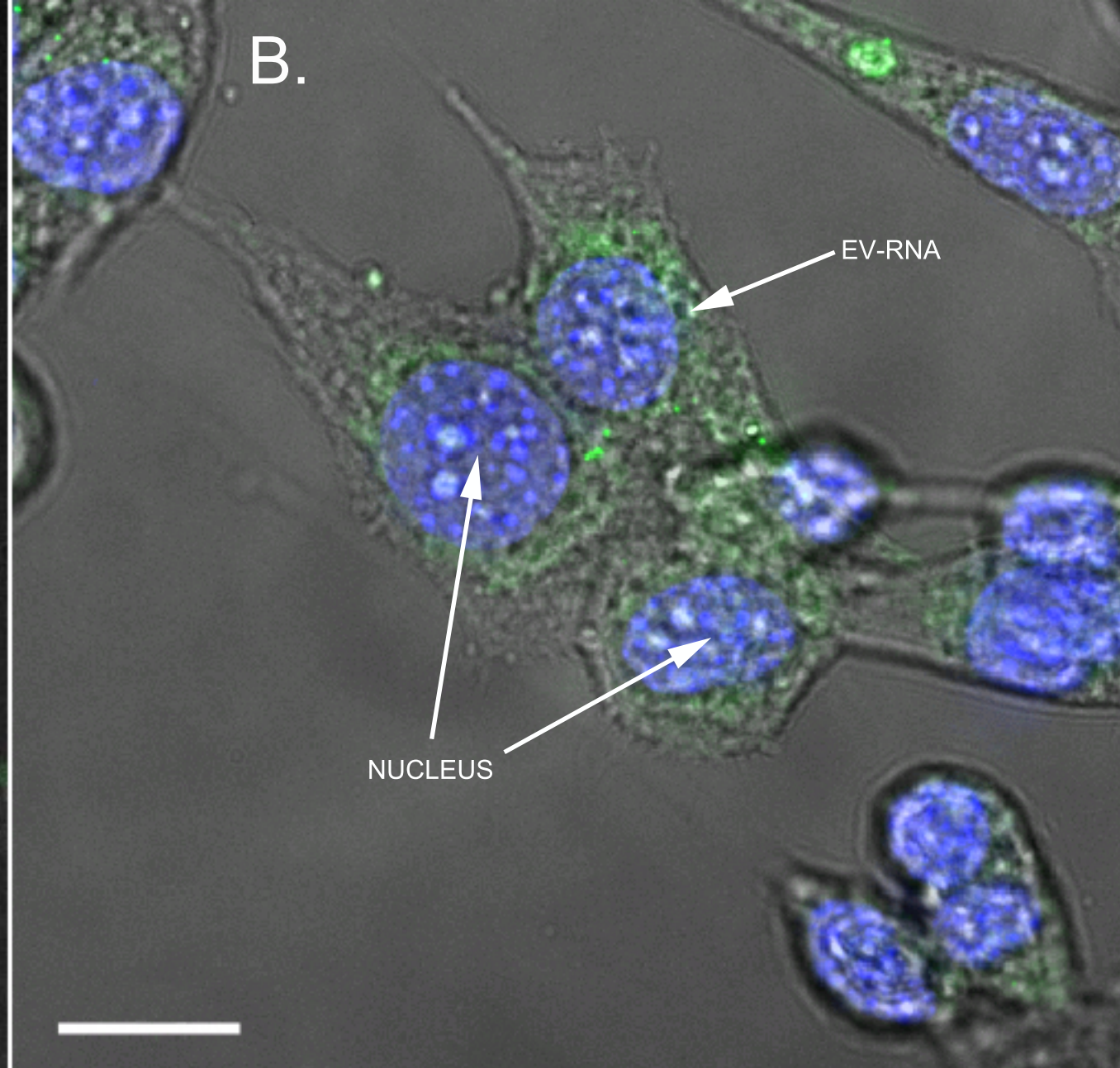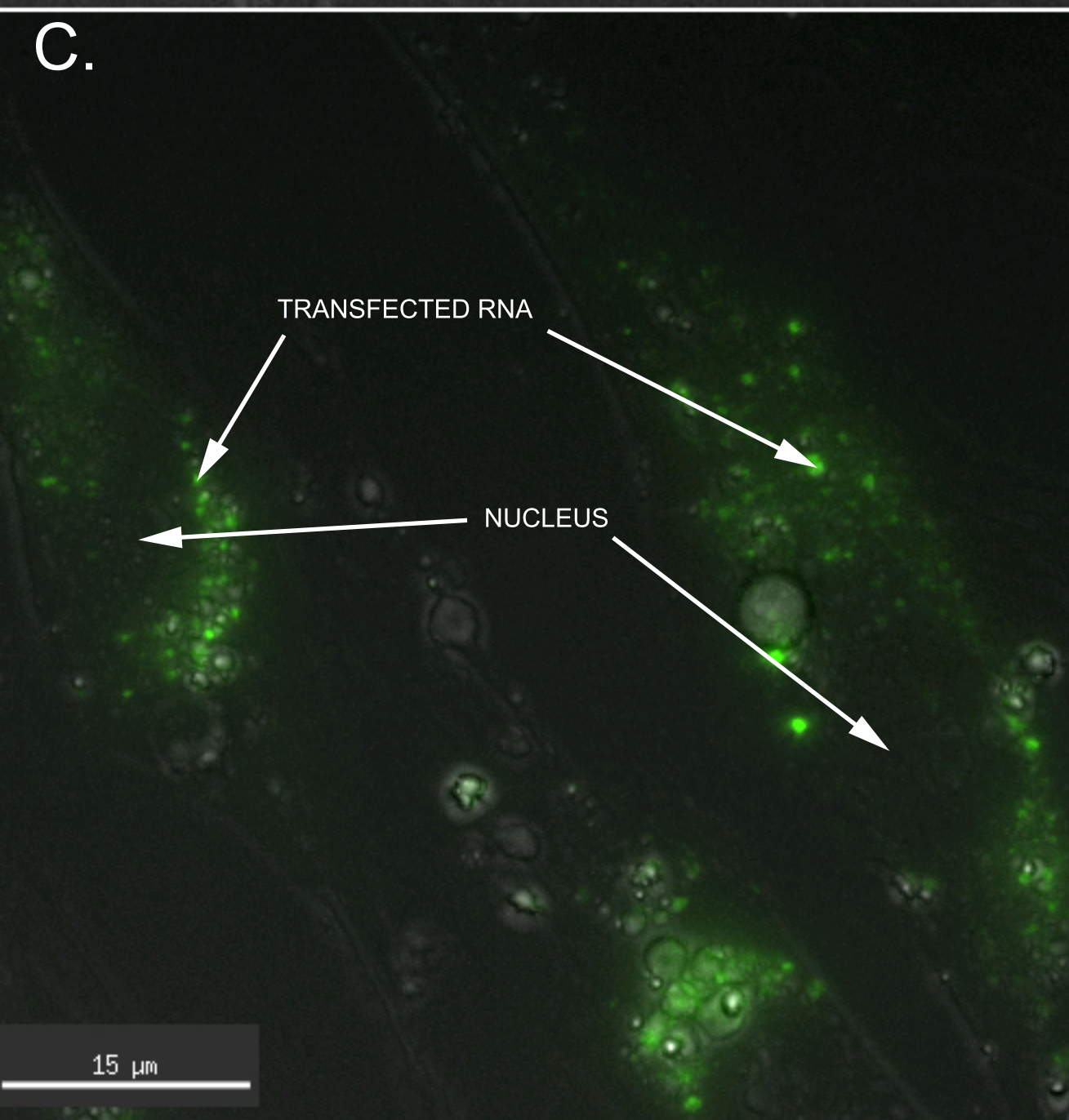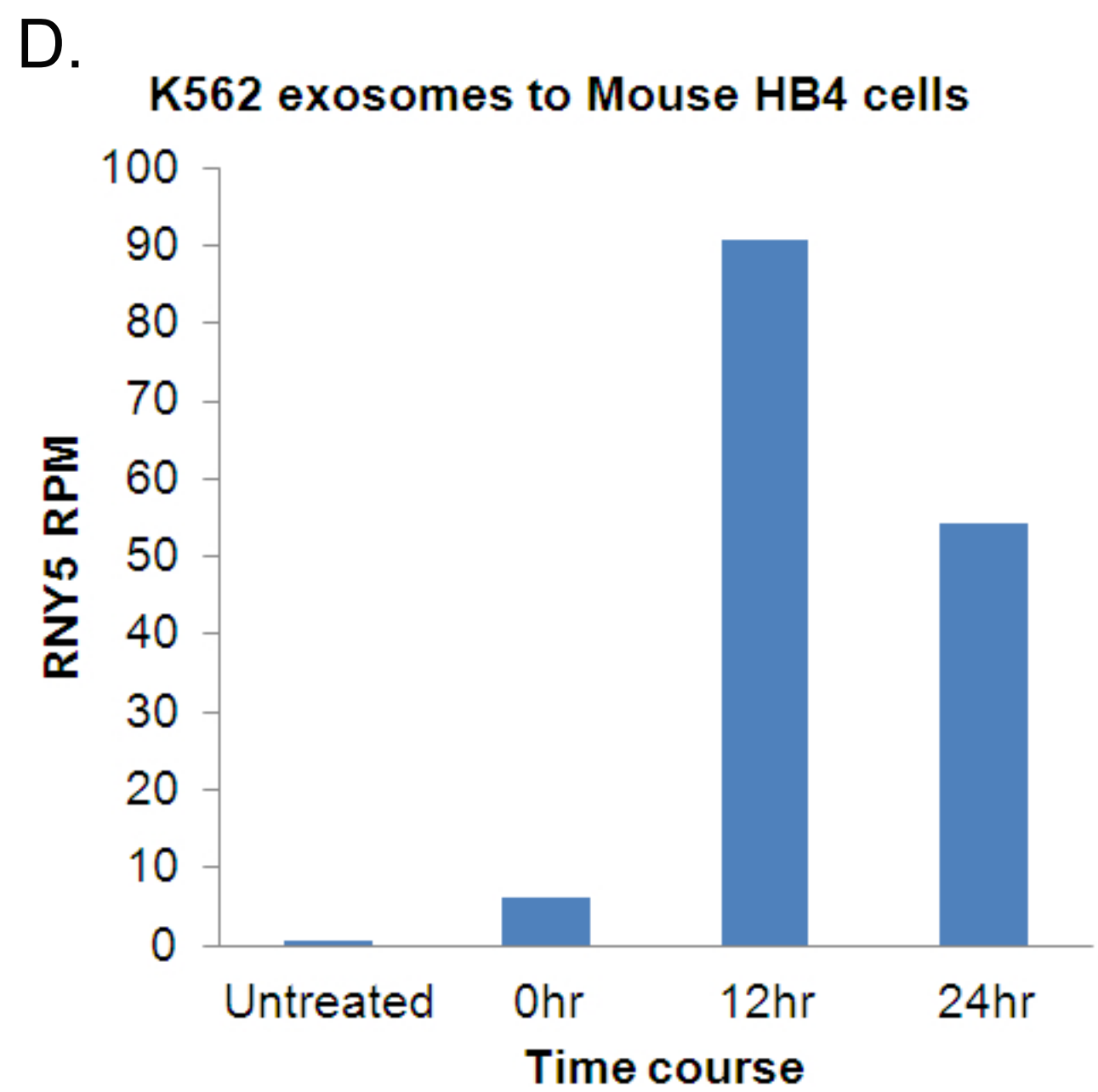

Supplement: Supplemental Material [file supp_053629.115_FigureS4.pdf]

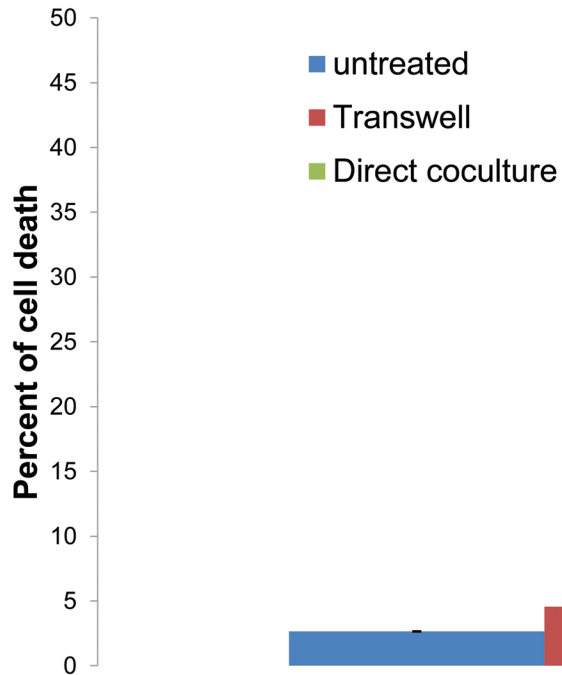

Supplement: Supplemental Material [file supp_053629.115_FigureS5.pdf]
