# Supplementary material for: Extracellular vesicle-mediated transfer of processed and functional RNY5 RNA
Source: RNA. 2015 Nov;21(11):1966–79. doi: 10.1261/rna.053629.115 (PMC4604435; doi:10.1261/rna.053629.115)
Supplement: Supplemental Material [file supp_053629.115_Table_S1.pdf]

Table S1. Number of EVs and total RNA yield (per 1+ E8 cells)

Cells	Number of EVs	Quantity of RNA
K562	1.135E+11	2-3ug
BJ	4.75E+09	800ng-1ug
