# Supplementary material for: Extracellular vesicle-mediated transfer of processed and functional RNY5 RNA
Source: RNA. 2015 Nov;21(11):1966–79. doi: 10.1261/rna.053629.115 (PMC4604435; doi:10.1261/rna.053629.115)
Supplement: Supplemental Material [file supp_053629.115_Table_S2.pdf]

TABLE S2: RNAseq mapping statistics

	K562 EV1	K562 EV2	K562 WC1	BJ EV1	BJ EV2	BJ WC1	BJ WC2
Number of input reads	15312204	38109015	37450624	12805596	13757050	13474063	18944518
Average input read length	28	38	60	30	43	62	55
UNIQUE READS:							
Uniquely mapped reads number	5021255	16183627	28362474	3821688	5881994	10658696	13182629
Uniquely mapped reads %	32.79	42.47	75.73	29.84	42.76	79.11	69.59
Average mapped length	38.12	49.03	63.5	33.06	56.73	63.26	57.73
MULTI-MAPPING READS:							
Number of reads mapped to multiple loci	3778069	11996007	5116446	6452579	5784261	1620129	3399157
% of reads mapped to multiple loci	24.67	31.48	13.66	50.39	42.05	12.02	17.94
UNMAPPED READS:							
% of reads unmapped	42.54	26.05	10.61	19.76	15.2	8.87	12.47
