# Supplementary material for: Extracellular vesicle-mediated transfer of processed and functional RNY5 RNA
Source: RNA. 2015 Nov;21(11):1966–79. doi: 10.1261/rna.053629.115 (PMC4604435; doi:10.1261/rna.053629.115)
Supplement: Supplemental Material [file supp_053629.115_Table_S3.pdf]

TABLE S3: Fold change in genes within TGFβ pathway after treatment with K562 EV/32mer

		BJ		HUVEC	
Ensembl ID	Gene Name	EV	Y5 32mer	EV	Y5 32mer
ENSG00000011485	PP5	2.976875179	3.421057404	0.0676865	0.0084047
ENSG00000026103	FAS	4.656635422	2.048862289	0.009357006	0.563608352
ENSG00000034152	MKK3	0.09353015	0.0445025	0.0338432	0.0275619
ENSG00000060656	PTP	3.061930644	0.326981718	0.0338432	0.02029715
ENSG00000080839	p107	0	0.0171034	0	1.876965838
ENSG00000081189	MEF2C	0.117922946	1.08374729	0.583742961	0.421850359
ENSG00000099942	CRKL	0.175407565	0.31541529	4.121847102	0.442392244
ENSG00000100393	p300	3.197199574	3.231212849	0.164448822	4.850764805
ENSG00000100614	PP2CA	2.339504222	0.51617598	0.093728919	0.582309083
ENSG00000105173	CycE	0.0531801	0	0	2.380874956
ENSG00000105329	TGFB	2.058540504	1.693291308	0.131157533	0.403729161
ENSG00000105810	CDK6	4.544841922	2.544864042	0.025786698	12.85967948
ENSG00000105851	PI3K	0	0	0.00216821	0.0202628
ENSG00000106799	TGFBR1	0.191907325	0.308684282	0.217953267	1.018430748
ENSG00000108984	MKK6	0	1.743880337	0	0.01867215
ENSG00000110092	CycD	7.03248404	4.04805578	0.013477673	0.154756127
ENSG00000110395	CBL	5.852682073	1.616081412	0.021747473	0.149827381
ENSG00000111276	KIP1	0.464566725	2.858382611	0.641712284	4.832878577
ENSG00000112062	p38	3.576198214	7.138180358	0.328897645	2.043336133
ENSG00000116717	GADD45	4.831559762	0.547287857	2.056499589	1.847321473
ENSG00000117560	FASL	0	0	0	0
ENSG00000120129	MKP	0.229483538	0.390175081	0.138351762	0.297863431
ENSG00000123080	INK4C	0.613554654	1.582814268	0.152010301	2.406647683
ENSG00000123374	CDK2	6.12384958	6.836905194	0.082224316	1.471357954
ENSG00000124762	CIP1	4.745429033	3.980018471	0.132035636	3.616877976
ENSG00000125952	MAX	0.434920714	0.406949119	1.025513794	0.692645795
ENSG00000129355	INK4D	0.0198651	0.02980155	0	0.02029715
ENSG00000129757	KIP2	0.194830207	0.577973107	0.04777165	0.190159
ENSG00000132646	PCNA	0.0198651	0.059603	0	0.02029715
ENSG00000133740	E2F5	0.497752197	0.433805695	0.015289917	0.22448795
ENSG00000135446	CDK4	3.166250701	0.666385609	0.019019883	0.308141895
ENSG00000136997	c-MYC	10.50665047	5.430016103	0.059701521	2.688282508
ENSG00000141510	p53	1.98187379	3.096556868	0.0313505	0.269983647
ENSG00000141646	SMAD4	0.555839525	1.600218374	0.089552557	2.991030495
ENSG00000142208	AKT	3.356141175	1.42663778	0.018674131	0.21442243
ENSG00000145386	CycA	0.545009408	0.877365545	0.036757445	0.186117826
ENSG00000147883	INK4B	0.167797249	0.182268818	0.046117279	1.966090745
ENSG00000147889	INK4A	0.222979975	1.687551851	0.038680165	0.222069396
ENSG00000150907	FKHR	4.826340311	0.435969251	0.095522544	0.411308598
ENSG00000163513	TGFBR2	8.577095271	3.259840411	4.335404091	5.448230223
ENSG00000166949	SMAD3	0.23328192	0.299832248	0.187844567	0.32353526
ENSG00000167193	CRK	0.463050572	0.343327532	0.09405179	0.146491557
ENSG00000168229	DP1	0.0198651	0.0218838	0	0.02521415
ENSG00000175197	GADD153	1.612330178	1.296914308	0.014444114	0.231295431
ENSG00000175387	SMAD2	0.341380875	0.658706903	0.087181307	0.87631948
ENSG00000185591	SP1	0.20910152	0.244202591	0.0676865	0.03534555
ENSG00000197442	ASK1	0.290445158	2.056268419	0.08161495	0.0275619
ENSG00000204209	DAXX	6.911299992	1.936330232	0.046970451	0.251868988
ENSG00000205250	E2F4	0.287780212	0.110088552	0.016827566	0.724578517
ENSG00000253729	DNA-PK	0.273054769	0.51968317	0.163465453	2.415904373
