# Supplementary material for: Extracellular vesicle-mediated transfer of processed and functional RNY5 RNA
Source: RNA. 2015 Nov;21(11):1966–79. doi: 10.1261/rna.053629.115 (PMC4604435; doi:10.1261/rna.053629.115)
Supplement: Supplemental Material [file supp_053629.115_Table_S4.pdf]

S4A. Percent cell death observed in K562 cells when treated with K562 EVs and EV RNA

Sample	Rep1	Rep2	Mean
Untreated	2.4	3.5	2.95
K562 EV treated	6.6	4.2	5.4
MOCK treated	4.4	4.3	4.35
K562 EV RNA treated	4.2	4	4.1
Complete scrambled 31-mer treated	3.5	4.5	4

S4B. Percent cell death observed in BJ cells when treated with cancer and primary EVs and EV RNA

Sample	Rep1	Rep2	Mean
Untreated	4	4.9	4.45
Mock treated	7.1	7.7	7.4
BJ EV RNA treated	10.3	10.9	10.6
K562 EV RNA treated	20	21	20.5
HELA EV treated	26.8	18.52	22.66
U2-OS EV treated	26.9	11.36	19.13
MCF7 EV treated	35.45	16.9	26.175
K562 EV treated	27.8	21.9	24.85
BJ EV treated	5.4	4.7	5.05

S4C. Net increase in cell death with 100 picomoles RNY5 31-mer treatment in primary and cancer cells (RNY5 treatment-Mock)

Cells	Rep1	Rep2	Mean
BJ	17.25	16.85	17.05
IMR90	8.9	9.4	9.15
HUVEC	14.7	13.5	14.1
HFFF (200picomoles RNY5 31-mer)	13.6	13.4	13.5
MCF7	0	0	0
HeLa	8.15	7.1	7.625
U2-Os	0.75	2.75	1.75
K562	0	2	1

S4D. Dose response (percent cell death) of RNY5 31-mer and Nonspecific RNA treatment in BJ cells

Sample	Rep1	Rep2	Mean
Untreated	1.3	1.6	1.45
Mock treated	2.6	2.5	2.55
Nonspecififc RNA 10picomoles	5.1	4.9	5
Nonspecific RNA 50picomoles	5.5	5.5	5.5
Nonspecific RNA 100picomoles	5.8	5.8	5.8
Nonspecific RNA 200picomoles	8.3	8.2	8.25
Nonspecific RNA 300picomoles	6.2	6.5	6.35
Nonspecific RNA 400picomoles	11.2	10.6	10.9
RNY5 31-mer10picomoles	6.4	6.5	6.45
RNY5 31-mer 50picomoles	8.8	9.1	8.95
RNY5 31-mer 100picomoles	12.2	12	12.1
RNY5 31-mer 200picomoles	23.8	22.3	23.05
RNY5 31-mer 300picomoles	30	29.6	29.8
RNY5 31-mer 400picomoles	40.9	40.5	40.7

S4E. Percent cell death in BJ cells with synthetic RNY5 31-mer and controls

Sample	Rep1	Rep2	Mean
Untreated	1.45	2.5	1.97
Mock treated	2.75	4	3.37
Allstar nonspecific RNA control	6	5.8	5.9
8 nucleotide motif deleted	5.3	4.8	5.05
Complementary side 32-mer	4.2	4.5	4.35
8 nucleotide motif scrambled	7.1	7.4	7.25
RNY5 completely scrambled 31-mer	6.6	7.9	7.25
Double stranded RNY5 31-mer	9.3	9.3	9.3
Full length RNY5	10.5	11	10.75
RNY5 31-mer	19.8	17.43	18.61
RNY5 23-mer	25.8	26.4	26.1

S4F. Percent cell death in K562 cells with synthetic RNY5 31-mer and controls

Sample	Rep1	Rep2	Mean
Untreated	9.2	10.5	9.85
Mock	9.7	10.5	10.1
8nt motif deleted	11.9	10.5	11.2
8nt motif scrambled	10.3	10.2	10.25
Allstar nonspecific RNA treated	11.6	9.7	10.65
31mer scrambled	8.9	8.8	8.85
Full length RNY5	9.8	9.5	9.65
Double stranded RNY5 31-mer	10.3	10.3	10.3
RNY5 31-mer	9.8	12.1	10.95
