# Supplementary material for: Extracellular vesicle-mediated transfer of processed and functional RNY5 RNA
Source: RNA. 2015 Nov;21(11):1966–79. doi: 10.1261/rna.053629.115 (PMC4604435; doi:10.1261/rna.053629.115)
Supplement: Supplemental Material [file supp_053629.115_SuppFig_Table_Legends.docx]

# Supplementary Figures

Figure S1. Isolation and quantification of EVs. S1A. Protocol for isolation of EVs from conditioned cell culture medium. S1B. Quantification and Size distribution of K562 EVs by Nanoparticle Tracking analysis (NTA). X-axis represents particles size (in nm) while Y-axis represents concentration of particles (E6)/ml.

Figure S2. Scatter plots representing correlation in gene expression levels, between replicates of EVs and cellular small RNA in K562 (Figure S2A) or BJ (Figure S2B).

Figure S3. Graphs depicting kernel density plots of the ratio of rpm in EV and the sum of rpm in EV and corresponding whole cell in K562 (Figure S3A) and BJ (Figure S3B). Each line in the plots depicts the number of genes belonging to each RNA family, and genes which have a ratio of 0 represents genes that are more abundant in cells compared to EVs, which a ratio of 1 represents genes that are more abundant in EVs when compared to their source cells.

Figure S4. Intercellular transfer and subcellular localization of EVs and EV-RNA.(A) Transfer and subcellular localization of K562 EVs labeled with lipid dye PKH67 in BJ cells. (B) Transfer and subcellular localization of 5-ethynyl uridine (EU) labeled K562 EV RNA (green) in Mouse 3T3 cells treated with ActinomycinD. Nuclei are counterstained with Hoechst33342. The scale bar represents 20microns. (C) Subcellular localization of synthetic RNY5-31mer labeled with Alexa-488 at 3’end in BJ cells after 24 hours. Scale bar indicates 15 microns. (D) Time course analysis of the level of RNY5 31-mer in mouse HB4 cells when Mouse HB4 cells are incubated with K562 EVs. X-axis indicates duration of incubation (hours) while Y-axis indicates the level of RNY5 (in reads per million).

Figure S5. Quantification of cell death of BJ cells by co-culture with K562. Y-axis indicates the percent cell death: (■) Untreated: BJ cells grown without any treatment, (■) Transwell: Percent cell death observed in BJ cells when co-cultured with K562 cells across a Transwell membrane (1micron pore size) at 1:1 ratio, (■) Direct co-culture: Percent cell death observed in primary BJ cells when BJ cells are directly co-cultured in the same well with K562 cells at 1:1 ratio.

# Supplementary Tables

Table S1. Number of EVs (quantified by Nanoparticle Tracking analysis) and quantity of RNA (quantified by Nanodrop) isolated from 1+E8 K562 and BJ cells.

Table S2. RNA sequencing statistics for all the different sequencing libraries from both K562 and BJ EVs and whole cells.

Table S3. Fold change in genes within the TGF-β pathway after treatment of BJ and HUVEC cells with EV derived from K562 and the synthetic 5’ 31 nucleotide fragment.

Table S4 (A) Percent of cell death observed in K562 cells when treated with K562 EV and EV RNA. (B) Percent of cell death observed in BJ cells when treated with cancer and primary EV and EV RNA. (C) Net increase in cell death with 100 pico-moles of RNY5 31mer treatment of cancer and primary cells (RNY5 treatment - mock). (D)Dose response of RNY5 31mer (percent cell death) and nonspecific RNA control in BJ cells. (E) Percent cell death in BJ cells with synthetic RNY5 31mer and controls. (F) Percent cell death in K562 cells with synthetic RNY5 31mer and controls.
